# Behavioral regulation in sport questionnaire and sport motivation scale-II: a scale comparison

**DOI:** 10.3389/fpsyg.2025.1652580

**Published:** 2025-09-11

**Authors:** Joana Bica, Miguel Jacinto, Rui Matos, Nuno Amaro, Raúl Antunes, Nuno Couto, Luís Cid, Pedro Forte, Diogo Monteiro

**Affiliations:** ^1^ESECS, Polytechnic University of Leiria, Leiria, Portugal; ^2^Research Centre in Sport, Health and Human Development (CIDESD), Covilhã, Portugal; ^3^Rio Maior School of Sport, Polytechnic of Santarém (ESDRM-IPSantarém), Rio Maior, Portugal; ^4^CI-ISCE-ISCE Douro, Penafiel, Portugal; ^5^Department of Sports Sciences, Instituto Politécnico de Bragança, Bragança, Portugal; ^6^Instituto Politécnico de Bragança, Research Centre for Active Living and Wellbeing (LiveWell), Bragança, Portugal; ^7^Sport Department, Higher Institute of Educational Sciences of the Douro, Penafiel, Portugal

**Keywords:** motivation, self-determination theory, sport motivation scale-II, behavioral regulation in sport questionnaire, self-determinant theory

## Abstract

**Introduction:**

This study compared the psychometric properties of two primary instruments for assessing sport motivation based on Self-Determination Theory: the Sport Motivation Scale-II (SMS-II) and the Behavioral Regulation in Sport Questionnaire (BRSQ).

**Methods:**

A cross-sectional analysis evaluated the scales’ internal consistency, factor structure, convergent and discriminant validity, and model fit, which required post-hoc modifications. Measurement invariance and adherence to the theoretical simplex pattern were also tested.

**Results:**

The BRSQ demonstrated generally acceptable reliability, while the SMS-II showed deficiencies in its introjected, external, and amotivation subscales. Both scales faced validity challenges in distinguishing adjacent motivational constructs. Although measurement invariance was supported, correlations deviated from the theoretical quasi-simplex pattern.

**Discussion:**

The BRSQ appears more robust, but neither scale is flawless. Researchers must select instruments aligned with their specific objectives and interpret scores cautiously due to these psychometric limitations. This underscores the need for refined tools to better capture the dynamic complexity of motivation in sports.

## Introduction

### Self-determination theory

Motivation is a central psychological factor in sports, influencing athletes’ participation, performance, resilience, and well-being (Deci and Ryan, 1985; Vallerand, 1997). Defined as the process that initiates, guides, and sustains goal-directed behaviors, it acts as the energy driving athletic actions (Rodrigues and Monteiro, 2021). Self-Determination Theory (SDT; Ryan and Deci, 2017) provides a framework for understanding these motivational dynamics, positing that humans naturally strive for growth and that intrinsic (self-driven) and extrinsic (instrumentally motivated) behaviors yield distinct performance and well-being outcomes. A key tenet of SDT is the internalization of norms and behaviors, which explains why extrinsic motivation is further categorized by its locus of causality (Deci and Ryan, 1985).

SDT also emphasizes the interaction between the individual and the environment, suggesting that the internalization process can be facilitated or hindered by contextual conditions. In this sense, the authors developed the concept of basic psychological needs to understand how the environment can promote internalization, as well as the maintenance of intrinsic motivation (Ryan and Deci, 2017), which are: autonomy (i.e., power to choose one’s behavior and control one’s activities); competence (i.e., ability to succeed in challenging tasks and achieve desired results); and relatedness (i.e., development of emotional bonds based on trust and mutual respect). If these three needs are satisfied, individuals tend to experience a higher quality of motivation, a greater sense of general well-being, and greater involvement and investment in healthy behaviors (Teixeira et al., 2022). However, if the NPB (Basic Psychological Needs) are frustrated/dissatisfied, the individual may lose functionality, becoming a passive being, or may even tend to have compensatory behaviors, and may adopt addictive or aggressive behaviors that arise from contexts of frustration (Ryan and Deci, 2017).

Organismic integration theory (OIT), one of the micro-theories of SDT, is particularly relevant in the present study, as it provides the conceptual framework for understanding the different types of motivation regulation and its internalization along the motivational continuum. This aspect is essential for comparing BRSQ and SMS-II, as both assess motivation in sport based on the principles of SDT, focusing precisely on the distinction between the various levels of motivational regulation, as offered by OIT.

### Organismic integration theory

To date, motivation has tended to consider it only as an amount of energy directed towards a certain behavior (Ryan and Deci, 2017). SDT has changed this paradigm by looking at motivation not only in terms of quantity, but by breaking down the quality of motivation according to the degree of self-determination, along a motivational continuum (Ryan and Deci, 2017). This ranges from amotivation (i.e., lack of regulation or lack of intention to act), through the least self-determined forms (external and introjected regulation), to the most self-determined forms (identified and integrated), until reaching intrinsic motivation.

In addition to the clear distinction between the different types of motivation, Ryan and Connell (1989) propose the existence of a quasi-simplex pattern, where the types of regulation that are, theoretically, closer on the continuum correlate highly and positively with each other, while those that are further apart have weaker correlations or are even negatively correlated with each other (Ryan and Connell, 1989).

In the field of physical exercise, there is already clear evidence that more autonomous regulations predict greater persistence, performance and well-being, while more controlled ones are associated with less positive effects. In fact, Teixeira et al. (2012) concluded that more autonomous forms of motivation are positively related to desirable exercise outcomes, while controlled forms presented mixed results, ranging from negative to null relationships, thus proving the theoretical framework.

### Assessment of the motivational continuum

Briere et al. (1995), following the structure of the quasi-simplex pattern proposed by Ryan and Connell (1989), formulated the first version of the “Sport Motivation Scale” in France, named “Echelle de Motivation dans les Sports” (Briere et al., 1995). In the same year, Pelletier et al. (1995) translated and validated the questionnaire into English, naming it the “Sport Motivation Scale.”

Although validated, the SMS presents several limitations. Mallett et al. (2007) highlighted the fact that the scale does not cover the entire continuum proposed by the SDT (Ryan and Deci, 2017), since it does not include integrated regulation. Furthermore, they consider that the three factors of intrinsic motivation are not empirically distinguishable, which may compromise the accuracy of the results (Mallett et al., 2007). Another underlying criticism was the semantics of the questions, considering that they could become confusing and misinterpreted, possibly due to problems in the translation of the original French version (Mallett et al., 2007).

Martens and Webber (2002) and Riemer et al. (2002) identified internal consistency problems in the SMS, particularly in factorial validity, indicating that the measurement model does not fit adequately. Pelletier et al. (1995), Raedeke and Smith (2001), Martin and Cutler (2002), and Vlachopoulos et al. (2000) also reported that the scale has low reliability, with internal consistency considered unacceptable.

As part of their criticism, Mallett et al. (2007) proposed a revised version of the SMS-6. This version also received some criticisms, namely the integration of items that included questions related to integrated regulation, which appear to have been based on other motivation scales based on the SDT (e.g., Motivation Towards the Environment Scale; Pelletier et al., 1998) and which overlapped with items that assessed identified and intrinsic regulation (Pelletier et al., 2013).

Lonsdale et al. (2008) add that the extrinsic motivation subscales were not always adequately related, concluding that the SMS does not guarantee reliability and validity. For these reasons, Lonsdale et al. (2008) developed a questionnaire that would fill the gaps in the pre-existing scales, the Behavioral Regulation in Sport Questionnaire (BRSQ). The authors proposed two versions.

The BRSQ-8, which includes three forms of intrinsic motivation, as initially proposed by Pelletier et al. (1995); and the BRSQ-6, which contains the same items, but assesses one general intrinsic motivation.

Initial validation of this questionnaire provided evidence on the reliability and factorial validity of the BRSQ items in elite and non-elite athlete populations, emphasizing the idea that it should be used specifically for use with competitive sports participants and not in other contexts (Lonsdale et al., 2008). Although some relationships among motivational regulation were in line with the quasi-simplex pattern, there was a lack of discrimination between external and introjected regulation in terms of their relationships with amotivation; the identified and integrated regulation subscales also had high correlations with intrinsic motivation; and there was no discrimination among the self-determined subscales. In short, although the BRSQ appears to assess the constructs of the SDT, it does not appear to discriminate between subscales assessing self-determined forms of motivation and subscales assessing non-self-determined forms of motivation (Lonsdale et al., 2008).

With the ongoing evolution of the SDT and based on concerns raised about the original SMS, Pelletier et al. (2013) proposed a reformulation of the scale. A panel of experts, including Edward Deci, Luc Pelletier, Robert Vallerand, and Richard Ryan, identified that some items on the scale were not sufficiently clear or aligned with the constructs defined by the SDT, which compromised its validity. As a result, these items were removed, and items were created to replace them. In addition, they decided to include the integrated regulation factor, essential to cover the entire continuum of motivation proposed by the SDT. Another change was the simplification of the assessment of intrinsic motivation. These changes aimed to improve the clarity, validity and consistency of the scale, ensuring its alignment with the principles of SDT, and so the ‘Sport Motivation Scale-II’ (SMS-II) was created.

This study aimed to compare the psychometric performance of the Sport Motivation Scale-II and Behavioral Regulation in Sport Questionnaire, scales under the Self-Determination Theory framework.

## Method

The comparison covers the psychometric properties of both instruments, including the assessment of reliability, discriminant and convergent validity. In addition, the adjustment indices of the models, such as CFI (Comparative Fit Index), TLI (Tucker-Lewis Index), RMSEA (Root Mean Square Error of Approximation) and SRMR (Standardized Root Mean Square Residual), are analyzed. The review also examines the invariance of the instruments in different groups and contexts. In addition, a meta-analysis was carried out with the purpose of exploring the effect of correlations along the motivational continuum proposed by the SDT.

### Information sources and research strategies

The search was carried out in English in the following electronic databases: Web of Science, Scopus and PubMed, accessed between June and September 2024, considering a time window between 2008 and 2024 for the BRSQ and 2013 and 2024 for the SMS-II. The search strategy combined the following terms: (“sport motivation scale” or “SMS-II”) AND (valid* OR “psychometric”) AND (motiv* OR regulat* OR behav*); (“behavioral regulation in sport questionnaire” or “BRSQ”) AND (valid* OR “psychometric”) AND (motiv* OR regulat* OR behav*). Studies cited in reference of the studies obtained in the databases were also considered.

### Eligibility criteria

For the selection of studies, the following inclusion criteria were considered: (i) studies published between 2008 (date of origin of the BRSQ) and 2024 and 2013 (date of origin of the SMS-II) and 2024; (ii) studies that validated the BRSQ or SMS-II; (iii) studies in English, Portuguese or Spanish; (iv) complete studies. Likewise, exclusion criteria were developed: (i) studies that only validated original SMS; (ii) studies presented in abstracts, letters to the editor.

### Data extraction process

The study was carried out independently by one researcher, who downloaded all the studies from the databases into the ENDNOTE X7 software, and duplicate studies were removed. The study selection process was conducted in sequential steps. In the first phase, potentially relevant studies were selected based on the analysis of titles and abstracts. If there was uncertainty about the relevance of a study, it was forwarded to the next phase. In the second phase, the pre-selected studies were assessed according to predefined eligibility criteria, ensuring that only those that met the requirements were included. Finally, a detailed analysis of the selected studies was performed, extracting all information and characteristics relevant to the review.

### Statistical analysis

The meta-analysis followed the guidelines established in the Preferred Reporting Items for Systematic Review and Meta-Analysis Protocols, by Moher et al. (2015). To carry out this, the statistical software R studio, version 4.2.3 (R Core Team, 2023) was used. The “metacor” function of the “meta” package was used for the meta-analysis of the r and r values of their respective number of participants. The overall correlations were calculated through the correlation and the number of participants in each study. The effect sizes were measured using the random effects model, with a 95% confidence interval (CI), magnitude of the effects and assessment of statistical significance (*p* < 0.05). Cohen’s effect size was classified considering the following ranges: d values between 0.2 and 0.5 were considered a small effect size, between 0.5 and 0.8 represented a medium effect size, and values greater than 0.8 indicated a large effect size (Cohen, 1988). Heterogeneity was analyzed using the X 2 and I 2 statistics, where a *p* value < 0.05 or and I 2 values > 50% indicate considerable heterogeneity (Higgins et al., 2003).

### Presentation of results

Based on the research carried out in the databases, a total of 133 studies were identified. Subsequently, after eliminating duplicates, reading the titles and abstracts and selecting them using previously defined eligibility criteria, a sample of 34 studies was created for complete analysis (17 related to SMS-II and 17 related to BRSQ).

### Participants

The review sample is defined based on the number of subjects in each validation of each instrument.

### Measurement techniques

The instruments were analyzed in several measures.

Reliability assesses the degree of consistency of the factors and this assessment is mainly made using Cronbach’s alpha, considering that values above 0.70 are generally considered acceptable (Nunnally, 1978). Additionally, McDonald’s omega (*ω*) was used in this analysis, with a cut-off value of 0.70 (Campo-Arias and Oviedo, 2008).

Convergent validity was analyzed considering Average Variance Extracted (AVE) values greater than 0.50. Discriminant validity is assessed by comparing the AVE of each factor with the square of the correlations of these same factors. Constructs are identified as distinct when the AVE value of each is greater than the value of the square of the correlation between these same constructs (Hair et al., 2019). Standardized factor weights are essential for interpreting the role of each item in the definition of each factor (Hair et al., 2019). For an item to explain at least 25% of the variability of a latent factor, its standardized factor weight must be at least 0.5.

Adjusting models to data, using confirmatory factor analysis, is essential to assess whether the model adequately represents the observed data (Byrne, 2016; Hair et al., 2019). When the adjustment values do not reach the acceptable limits, the model does not adequately represent the observed data, and there may be a restructuring of the models to improve the adjustment indices (Byrne, 2016; Kline, 2016). However, there must always be new validation with an independent sample to ensure the adjustment to new data (Byrne, 2016). Also, the model invariance was checked.

## Results

Appendix A correspond to the studies and respective characteristics of the analyzed validations of SMS-II and BRSQ, respectively.

In the SMS-II validations, the geographic diversity was quite diverse. Sample sizes ranged from 25 (Li et al., 2016) to 1,197 athletes (Smohai et al., 2021). All studies present a balanced distribution between men and women and mean ages range from 13.64 (Granero-Gallegos et al., 2018) to 40.44 (Pelletier et al., 2013), with most studies focusing on adolescents and young adults. The studies include both high-performance athletes (Pineda-Espejel et al., 2016) and athletes from secondary schools (Granero-Gallegos et al., 2018) or universities (Li et al., 2016).

Regarding the BRSQ, the studies also vary in their geographical area, with participants from several countries. The sample sizes also vary widely, ranging from 34 (Lonsdale et al., 2008) to 7,769 athletes (Viladrich et al., 2013). In most cases, the distribution between men and women was equivalent, with the exception of the study by Shokri et al. (2014). The average age ranged from 11.76 (Viladrich et al., 2013) to 28.65 (Shokri et al., 2014). The studies include different levels of athletes, from young football players (Viladrich et al., 2013) to professional elite athletes (Lonsdale et al., 2008).

Both studies cover a diverse range of team and individual sports.

Appendix B contain the standardized factor weights, reliability values, convergent validity values and information about discriminant validity, SMS-II and BRSQ, respectively. In addition, Supplementary material contains tables summarizing the number of studies reporting each of the problems.

### Reliability

#### Sport motivation scale-II

Of the 17 validations, 16 included the assessment of factor reliability, with the majority of studies (Barreira et al., 2022; Chin et al., 2021; Granero-Gallegos et al., 2018; Jelínek et al., 2021; Li et al., 2016; Nascimento et al., 2014; Pelletier et al., 2017; Pereira et al., 2024; Pineda-Espejel et al., 2016; Smohai et al., 2021; Vallejo-Reyes et al., 2018; Viciana et al., 2017) identifying reliability problems, mainly in introjected and external regulation and amotivation, that is, below 0.70.

Introjected regulation was reported with values below the threshold in nine studies, having presented worryingly low values, such as *α* = 0.32 (Barreira et al., 2022) and α = 0.39 (Vallejo-Reyes et al., 2018), indicating weak internal consistency. External regulation and amotivation also presented relatively low values in six of the studies analyzed, each.

The intrinsic, integrated and identified regulation dimensions presented fewer studies identifying values below 0.70. Even those that do not reach this value vary between 0.63 and 0.69.

#### Behavioral regulation in sport questionnaire

Of the 17 validations, 14 included the assessment of the reliability of the factors, with only five studies (Shokri et al., 2014; Monteiro et al., 2018; Moreno-Murcia et al., 2011; Tsitskari et al., 2015; Viladrich et al., 2011) reporting reliability problems. The values presented in these studies appear in integrated, identified, external and amotivation regulations and vary between α = 0.61 and α = 0.68.

### Convergent validity

One validation of the SMS-II and one of the BRSQ did not present AVE values or standardized factor weight values that could be used to calculate the AVE value (Barreira et al., 2022 and Filippos et al., 2019, respectively). However, although both scales present several AVE values <0.50, not all of them represent a lack of convergent validity. If all items of the factor in question present standardized factor weights >0.50, we can assume that the factors have adequate convergent validity (Hair et al., 2019).

#### Sport motivation scale-II

Several studies (Granero-Gallegos et al., 2018; Chin et al., 2021; Jelínek et al., 2021; Li et al., 2016; Nascimento et al., 2014; Pelletier et al., 2013; Pineda-Espejel et al., 2016; Stenling et al., 2015; Vallejo-Reyes et al., 2018; Viciana et al., 2017) identified AVE values <0.50.

Neither intrinsic nor integrated regulation showed issues with convergent validity. Regarding identified regulation, only Vallejo-Reyes et al. (2018) lacked convergent validity. Introjected regulation had six studies with convergent validity problems (Nascimento et al., 2014; Pineda-Espejel et al., 2016; Viciana et al., 2017; Vallejo-Reyes et al., 2018; Jelínek et al., 2021; Pereira et al., 2024). External regulation and amotivation showed a lack of convergent validity in three studies each (Stenling et al., 2015; Chin et al., 2021; Jelínek et al., 2021 for external regulation; and Stenling et al., 2015; Li et al., 2016; Chin et al., 2021 for amotivation).

#### Behavioral regulation in sport questionnaire

Several studies (Cece et al., 2019; Hancox et al., 2015; Lonsdale et al., 2008; Monteiro et al., 2018; Monteiro et al., 2019; Moreno-Murcia et al., 2011; Shokri et al., 2014; Stenling et al., 2018; Tsitskari et al., 2015; Viladrich et al., 2011; Viladrich et al., 2013) identified AVE values <0.50.

Intrinsic regulation and identified regulation showed a lack of convergent validity in one study each (Cece et al., 2019; Shokri et al., 2014, respectively).

The identified regulation has five studies confirming its lack of convergent validity (Cece et al., 2019; Monteiro et al., 2018; Moreno-Murcia et al., 2011; Tsitskari et al., 2015; Viladrich et al., 2013).

Regarding introjected regulation, three confirmed the lack of convergent validity (Viladrich et al., 2013; Cece et al., 2019; Stenling et al., 2018). External regulation, in turn, verified its lack of convergent validity in two studies (Viladrich et al., 2013; Tsitskari et al., 2015) and amotivation in one (Tsitskari et al., 2015).

### Discriminant validity

#### Sport motivation scale-II

SMS-II validations revealed problems in several combinations of factors, such as intrinsic-integrated (7), intrinsic-identified (5), intrinsic-introjected (3), integrated-identified (7), integrated-introjected (5), integrated-external (1), identified-introjected (4), introjected-external (1). Of the 17 studies analyzed, three did not contain data on discriminant validity (Barreira et al., 2022; Smohai et al., 2021; Vallejo-Reyes et al., 2018) and six did not report problems (Baaziz et al., 2023; Paic et al., 2017; Pelletier et al., 2013; Pelletier et al., 2017; Pineda-Espejel et al., 2016; Rodrigues et al., 2021).

#### Behavioral regulation in sport questionnaire

Four studies did not contain data (Cece et al., 2019; Çetinkaya and Mutluer, 2018; Filippos et al., 2019; Tsitskari et al., 2015) and one (Hancox et al., 2015) did not present discriminant validity issues. Of the problems found in the BRSQ, the most reported were between integrated-identified (7), introjected-external (7) and external-amotivation (6).

### Standardized factor weights

#### Sport motivation scale-II

Of the 17 validations analyzed in this study, two did not evaluate standardized factor weights (Barreira et al., 2022; Smohai et al., 2021). Of the remaining 15, only five (Baaziz et al., 2023; Granero-Gallegos et al., 2018; Paic et al., 2017; Pelletier et al., 2017; Rodrigues et al., 2021) presented all items with standardized factor weights ≥0.5.

#### Behavioral regulation in sport questionnaire

Of the 17 validations analyzed, one did not evaluate the standardized factor weights (Filippos et al., 2019). Of the remaining 16, nine (Alexe et al., 2022; Çetinkaya and Mutluer, 2018; Francisco et al., 2019; Guedes et al., 2019; Hancox et al., 2015; Lonsdale et al., 2008; Luo et al., 2023; Monteiro et al., 2019; Viladrich et al., 2011) presented all items with standardized factor weights ≥0.5.

### Adjustment indexes

#### Sport motivation scale-II

Baaziz et al. (2023), Granero-Gallegos et al. (2018), Li et al. (2016), Pelletier et al. (2013), Pelletier et al. (2017), Pineda-Espejel et al. (2016), Rodrigues et al. (2021) and Viciana et al. (2017) obtained adequate adjustment indices with the original model. Smohai et al. (2021) obtained adequate adjustment indices with the original model in the sample of recreational athletes, but not in the competitive ones (TLI = 0.89).

Barreira et al. (2022), Chin et al. (2021), Nascimento et al. (2014), and Pereira et al. (2024) did not obtain an adequate adjustment value with the original model, however, by correlating standard errors, they managed to improve the adjustment values. However, they have not validated the restructured model with an independent sample and cannot guarantee that the data captured will be applied to new data in the future.

Pineda-Espejel et al. (2016) and Viciana et al. (2017), although they obtained adequate adjustment values with the original model, tested alternative models. The first found that one of the introjected regulation items had a low standardized factor weight, and the model without this item was validated. It should be noted that this restructured model was not revalidated with an independent sample. The second, due to the lack of convergent validity of introjected regulation, eliminated this factor, tested and validated this model with better adjustments than the original. Even so, both studies faced the same problems: the fact that they were unable to clearly distinguish intrinsic, integrated and identified regulations, as well as introjected and external regulations. Therefore, following what the authors of SDT proposed (Ryan and Deci, 2017), they chose to test a model with global factors of motivation (autonomous and controlled) and amotivation. While Pineda-Espejel et al. (2016) obtained equally acceptable adjustment values with the original model, Viciana et al. (2017) did not (CFI = 0.78, TLI = 0.73, RMSEA = 0.09).

Paic et al. (2017), due to difficulties during the translation and validation process of the original SMS-II scale due to the similarity of meanings in the Hungarian version of the intrinsic and identified regulation factors, chose to translate all the items used in the formulation of the SMS-II and, subsequently, reduce them, forming a scale of six factors and 19 items. However, the authors believe that intrinsic motivation can be divided into motivation for development in sports and for technical learning. Thus, they created a second model in which they subdivided the intrinsic regulation: “cognitive intrinsic” and “effective intrinsic.” Although both models met the adjustment criteria, the second model presented better values. Furthermore, a comparison was made between the responses for the intrinsic subdimensions, justifying the distinction between effective and cognitive learning as different subfactors of intrinsic motivation.

It is important to note that both models that did not obtain adjustment in the first instance, as well as alternative models that were not retested with independent samples, deserve to be reviewed and, when used, the data must be analyzed with caution.

#### Behavioral regulation in sport questionnaire

Lonsdale et al. (2008) formulated and validated the BRSQ-6 and BRSQ-8 models with adequate adjustment indices. Guedes et al. (2019), similarly tested the two models with adequate adjustment indices.

Alexe et al. (2022), Çetinkaya and Mutluer (2018), Hancox et al. (2015), Luo et al. (2023), Monteiro et al. (2019) and Viladrich et al. (2011) tested the original BRSQ-6 model and obtained adequate adjustment values. With the BRSQ-6, Francisco et al. (2019) created a model reduced to two items per factor with adequate adjustment indices. In turn, Filippos et al. (2019) tested a nine-factor model in which, in addition to including general intrinsic regulation, it includes the three subdivisions of intrinsic regulation and obtained adequate adjustment indices.

In the original validation, Lonsdale et al. (2008) obtained high correlations between external and introjected regulation and between identified and integrated regulation. In this sense, different alternative models were tested, all without acceptable values: one with a single factor of controlled extrinsic motivation (introjected and external); one with single factor of autonomous extrinsic motivation (identified and integrated); one that’s compiling external, introjected regulation and amotivation into a single factor; one that’s compiling integrated, identified regulation and intrinsic motivation.

Viladrich et al. (2011), like the original authors of the scale (Lonsdale et al., 2008), tested alternative models, both without acceptable adjustment values; one with intrinsic motivation, autonomous extrinsic motivation, controlled extrinsic motivation and amotivation; one with intrinsic motivation, extrinsic motivation and amotivation.

Similarly, Hancox et al. (2015) tested alternative models, justifying their need by distinguishing between intrinsic and extrinsic motivation and autonomous and controlled motivation by Ryan and Deci (2000): M2 considers intrinsic motivation, autonomous extrinsic, controlled extrinsic and amotivation; M3 considers intrinsic motivation, extrinsic motivation and amotivation, as also tested by Viladrich et al. (2011); M4 consider autonomous motivation, controlled motivation and amotivation. Of these, both M2 and M4 obtained adequate adjustment values, however, only M2 presents ΔCFI <0.01 in relation to the original model. To further explore the issues of discriminant validity between integrated and identified regulations and between introjected and external items, two other alternative models were tested. The objective was to rule out the possibility that some items did not have a second-order structure and, instead, loaded directly on a first-order fact. Both had adequate adjustment values, however, only one (intrinsic, integrated, identified regulation, introjected/external items and amotivation) presents ΔCFI <0.01 with the original model. This model was also analyzed by the authors of the original scale (Lonsdale et al., 2008).

Shokri et al. (2014), who did not obtain adequate adjustment with the original model (RMSEA = 0.09), tested a model tested by the original authors of the scale (Lonsdale et al., 2008) that maintained intrinsic motivation, integrated and identified regulation, but contained a single factor with introjected, external regulation and amotivation, without adequate adjustment values (RMSEA = 0.09).

Monteiro et al. (2018) did not obtain adequate adjustment values, with the TLI value lower than expected (0.89) in the BRSQ-6. They tested a model composed of autonomous motivation and controlled motivation (six factors/24 items) and a similar model, but without amotivation (five factors/20 items). Without adequate adjustment.

Alexe et al. (2022) obtained adequate adjustment values with a model composed of autonomous, controlled motivation and amotivation, previously tested by other authors (Hancox et al., 2015; Monteiro et al., 2018).

Luo et al. (2023) also tested several models: M1 with six factors; M2 with amotivation, autonomous motivation and controlled motivation; M3 with two general motivation factors and six specific motivation factors; M4 with two general motivation factors, amotivation, external regulation and introjected regulation; M5 with two general motivation factors, amotivation, identified regulation, integrated regulation and intrinsic motivation; M6 with amotivation, controlled motivation, identified regulation, integrated regulation and intrinsic motivation; M7 with amotivation, external regulation, introjected regulation and autonomous motivation. Only the M1, M3 and M7 obtained adequate adjustment values. However, in M1 identified, integrated and intrinsic regulation were highly correlated. M3 revealed that the addition of a general autonomous motivation factor explained the covariance of the identified, integrated and intrinsic regulation factors, indicating that they could be part of the same factor. The same happens for the items of the external and introjected regulation factors, with the addition of a single controlled motivation factor. M7 was the model with the best adjustment, best standardized factor weights, score reliability estimates without extremely high factor correlations and was chosen as the best model. However, since there was no need to use the 12 autonomous motivation items, a model was created with the M7 factors, but with only four items in autonomous regulation. This final model obtained excellent adjustment indices.

Cece et al. (2019), Stenling et al. (2018) and Viladrich et al. (2011) also tested an alternative model, in which they did not include integrated regulation, because Vallerand (1997) states that it is not visible until reaching adulthood. While Cece et al. (2019) and Stenling et al. (2018) obtained adequate adjustment indices, Viladrich et al. (2011) did not (CFI = 0.89 and TLI = 0.87).

Moreno-Murcia et al. (2011) did not obtain an adequate adjustment value with the original BRSQ-8, however, by correlating standard errors, they managed to improve the adjustment values. However, they have not performed a validation with the restructured model, for an independent sample, not being able to guarantee that the captured data would be applied to new future data. Furthermore, Tsitskari et al. (2015) tested the BRSQ-8 model without adequate adjustment values (CFI = 0.58, TLI = 0.56). They created an alternative model with five factors (general intrinsic regulation, intrinsic regulation to experience, autonomous regulation, introjected regulation and external regulation), however, they did not perform confirmatory factor analysis to verify the model’s adjustment to the data.

### Measurement invariance

#### Sport motivation scale-II

Pelletier et al. (2013) confirmed that the SMS-II factor structure is invariant between men and women (Δx^2^*p* > 0.05), but the comparison between age groups (below and above 40 years) indicated a significant difference (Δx^2^*p* = 0.04), which compromised the assumption of invariance between age groups.

In the studies by Nascimento et al. (2014) and Pereira et al. (2024), the factor structure also showed invariance between men and women (Δx^2^*p* > 0.05), and temporal stability was confirmed after 7 days. Rodrigues et al. (2021), in addition to invariance between men and women, also found invariance between team and individual sports (ΔCFI < 0.01).

Granero-Gallegos et al. (2018) and Li et al. (2016) also found metric invariance between men and women (ΔCFI < 0.01), as well as between age groups (juniors and seniors) (Li et al., 2016).

Viciana et al. (2017) observed that the metric invariance of the SMS-II was not confirmed between men and women (Δx^2^*p* < 0.05), while the scalar invariance was maintained (Δx^2^*p* > 0.05). However, between federated and non-federated athletes, the metric invariance was verified (Δx^2^*p* > 0.05), but the scalar invariance was not (Δx^2^*p* < 0.05).

Baaziz et al. (2023) evaluated the temporal stability of the SMS-II over 4 weeks, confirming invariance over time.

#### Behavioral regulation in sport questionnaire

Guedes et al. (2019), Hancox et al. (2015), Lonsdale et al. (2008), Luo et al. (2023) and Monteiro et al. (2019) found that the structure of the BRSQ is invariant between men and women (∆CFI < 0.01).

Hancox et al. (2015) also confirmed the invariance between different levels of involvement with dance (recreational and vocational) and ages (<18 years and >18 years). Alexe et al. (2022) and Guedes et al. (2019) also did so for specific age groups (18–21 years and 22–52 years; 14–15 years and 16–17 years, respectively).

Luo et al. (2023) found invariance between different sporting levels (competition and professional), Alexe et al. (2022), between collective and individual sports and Monteiro et al. (2019) between different modalities.

Cece et al. (2019) and Stenling et al. (2018) proved the temporal invariance of the model (∆CFI < 0.01). Viladrich et al. (2013) proved the invariance of the model validated among young people from five European countries (∆CFI < 0.01).

### Effect of correlations on the motivational continuum

Table D.7 presents data relating to the meta-analysis that reveals the effect of correlations along the motivational continuum.

To carry out this analysis, only studies that contained the 15 correlations were considered. From SMS-II, the studies considered were: Baaziz et al. (2023); Chin et al. (2021); Granero-Gallegos et al. (2018); Jelínek et al. (2021); Nascimento et al. (2014); Li et al. (2016); Paic et al. (2017); Pelletier et al. (2013); Pelletier et al. (2017); Pereira et al. (2024); Pineda-Espejel et al. (2016); Rodrigues et al. (2021); Smohai et al. (2021); Stenling et al. (2015); Viciana et al. (2017). From BRSQ: Alexe et al. (2022); Francisco et al. (2019); Guedes et al. (2019); Hancox et al. (2015); Lonsdale et al. (2008); Luo et al. (2023); Monteiro et al. (2018); Monteiro et al. (2019); Moreno-Murcia et al. (2011); Shokri et al. (2014); Viladrich et al. (2011).

The two tables are in agreement in most correlations, and both are in agreement with the quasi-simplex pattern of the SDT, with some exceptions: intrinsic-introjected; integrated-external; identified-external. In these cases, the BRSQ shows negative values, in line with the SDT, while the SMS-II shows positive values. Both scales contradict the SDT in the integrated-introjected combination, which, theoretically, should be a negative.

More detailed correlations between each pair of factors and the respective studies can be found in Supplementary material.

### Heterogeneity

In all analyses performed, heterogeneity is a significant factor, highlighting the diversity and variability of the data observed. This characteristic reflects the inherent complexity of the groups analyzed, highlighting the need for methodological approaches that take this diversity into account to ensure representative conclusions. The presence of heterogeneity also reinforces the importance of a critical and contextualized analysis, allowing us to understand the nuances and particularities that influence the results obtained.

## Discussion

This study aimed to conduct a comparative analysis of two widely used scales for assessing motivation in sport, both based on the Self-Determination Theory (Ryan and Deci, 2017): Behavioral Regulation in Sport Questionnaire and Sport Motivation Scale-II. Not only to provide a detailed comparison between the instruments, but also to generate knowledge that may contribute to the advancement of research in sport motivation and to a more effective application of these scales in scientific and practical contexts.

### Reliability

Comparing the reliability between the two instruments, the SMS-II presents a more critical panorama in relation to the internal consistency of its dimensions, especially in the subscales of introjected regulation (Barreira et al., 2022; Chin et al., 2021; Li et al., 2016; Nascimento et al., 2014; Pereira et al., 2024; Pineda-Espejel et al., 2016; Smohai et al., 2021; Vallejo-Reyes et al., 2018; Viciana et al., 2017), external (Barreira et al., 2022; Chin et al., 2021; Nascimento et al., 2014; Smohai et al., 2021; Viciana et al., 2017) and amotivation (Barreira et al., 2022; Chin et al., 2021; Granero-Gallegos et al., 2018; Nascimento et al., 2014; Pineda-Espejel et al., 2016; Vallejo-Reyes et al., 2018; Viciana et al., 2017), which frequently do not reach the 0.70 threshold (Table B.3). Introjected regulation presents the worst reliability results, ranging from 0.32 (Table B.3, Barreira et al., 2022) to 0.69 (Table B.3, Li et al., 2016) in the SMS-II, indicating serious difficulties in measuring this construct consistently. As it is a partially internalized type of regulation (Ryan, 1993), it is more susceptible to contextual and individual variations. Furthermore, for the same reason, it can be difficult to distinguish it from other forms of regulation close to it on the continuum (identified and external). This conceptual ambiguity can cause semantic overlaps, resulting in low internal consistency (Streiner, 2003).

The BRSQ presents items formulated in a clearer and more precise manner, facilitating respondents’ interpretation. In SMS-II, the items appear to be more generic, with a semantic overlap between the types of regulation, especially in the factors related to internalization (Lonsdale et al., 2008). The items of these factors are so similar, both in meaning and in wording, that respondents can interpret them in a similar way, even though they theoretically represent different constructs (Vallejo-Reyes et al., 2018). This semantic overlap can lead to inconsistent responses (Streiner, 2003), especially in subscales such as introjected regulation and external regulation, since they are less internalized and, therefore, more susceptible to contextual and individual variations (Ryan and Deci, 2017). Amotivation, as it represents the absence of motivation, seems to be difficult to capture in a reliable way, since each person can interpret their lack of motivation in different ways, or even deny its existence (Ryan and Deci, 2017).

The low internal consistency of the SMS-II in relation to the BRSQ may also be due to the reduced number of items per factor (Pineda-Espejel et al., 2016), since most studies used Cronbach’s alpha value, which depends directly on the number of items per factor, that is, it ends up assuming that each item has the same importance for the respective factor, without considering the weight that each item actually has in the respective factor (Raykov, 1997).

### Validity

In several validations of the SMS-II, problems of convergent validity were revealed, particularly in the dimensions of introjected regulation (e.g., Chin et al., 2021; Granero-Gallegos et al., 2018; Jelínek et al., 2021; Li et al., 2016; Nascimento et al., 2014; Pereira et al., 2024; Pineda-Espejel et al., 2016; Vallejo-Reyes et al., 2018; Viciana et al., 2017), external (e.g., Chin et al., 2021; Granero-Gallegos et al., 2018; Jelínek et al., 2021; Nascimento et al., 2014; Stenling et al., 2015; Viciana et al., 2017) and amotivation (e.g., Chin et al., 2021; Granero-Gallegos et al., 2018; Li et al., 2016; Nascimento et al., 2014; Pereira et al., 2024; Stenling et al., 2015; Viciana et al., 2017), whose items do not appear to be measuring the same construct consistently. The BRSQ presents a less critical situation, with convergent validity problems being less pronounced in general, except for the identified regulation.

As far as introjected regulation is concerned, several authors attribute its lack of convergent validity to the semantic construction of the items in this factor, since they include items of approach and avoidance, affecting the convergence of the items (Nascimento et al., 2014; Viciana et al., 2017; Vallejo-Reyes et al., 2018). Viladrich et al. (2013) also identified as problematic the fact that the items of this regulation present cross-loading with the identified and external regulations, which may be related to the different approach and avoidance, respectively (Assor et al., 2009).

The AVE values in the BRSQ, compared to the SMS-II, are more consistent, indicating that the BRSQ may be more robust in terms of convergent validity. These differences can be explained not only by the semantic construction of the items, reported in introjected regulation (Nascimento et al., 2014; Vallejo-Reyes et al., 2018), but also by translation issues of the instrument (Barreira et al., 2022; Chin et al., 2021; Li et al., 2016), since there may be a loss of meaning or misinterpretation of the items (Chin et al., 2021; Li et al., 2016).

The SMS-II presents a larger overlap, for the most part, between autonomous regulations (as shown in Table B.3). This overlap seems to be justified by the items of these same factors that, despite being in factors that would theoretically be distinct, share similar semantic content, causing them to be interpreted as measuring the same underlying construct (Jelínek et al., 2021; Li et al., 2016; Nascimento et al., 2014). The items of the most autonomous factors of motivation regulation seem to be empirically indistinguishable, justifying the high correlations and problems of discriminant validity. Howard et al. (2017) concluded that intrinsic, identified and integrated regulations are situated extremely close on the self-determination continuum, which may create confusion between these factors, corroborating the justification for this overlap of factors. In addition to being extremely close, it appears that integrated regulation occupies a conceptual space that significantly overlaps with identified regulation and intrinsic motivation. By presenting these high correlations with both, it demonstrates a lack of differentiation, leading to problems of multicollinearity and difficulties in interpreting the results (Howard et al., 2017).

The integrated-identified combination was widely reported at both scales (Alexe et al., 2022; Granero-Gallegos et al., 2018; Jelínek et al., 2021; Li et al., 2016; Lonsdale et al., 2008; Luo et al., 2023; Monteiro et al., 2018; Monteiro et al., 2019; Moreno-Murcia et al., 2011; Nascimento et al., 2014; Pereira et al., 2024; Shokri et al., 2014; Stenling et al., 2015; Viciana et al., 2017). These constructs do not appear to be empirically distinguishable, which may be partly justified by their proximity on the continuum (Howard et al., 2017; Ryan and Connell, 1989). This same justification also seems to be plausible for the introjected-external and external-amotivation combinations, widely reported in the BRSQ (Alexe et al., 2022; Guedes et al., 2019; Lonsdale et al., 2008; Monteiro et al., 2018; Monteiro et al., 2019; Moreno-Murcia et al., 2011; Stenling et al., 2018; Viladrich et al., 2011, 2013).

The identified-introjected combination seems to have a more complex justification. Introjected regulation can be subdivided into two factors: introjected regulation by avoidance and by approximation (Assor et al., 2009). In both scales, the introjected regulation factor is composed of introjection items by avoidance and by approximation, which can cause confusion in distinguishing the items of the introjected regulation factor from those of identified regulation (Teixeira et al., 2022). Assor et al. (2009) also suggest that, in scales that assess motivation, there should be a separation of the introjected regulation factor, in its two forms, so that there is no overlap with identified regulation. Furthermore, it is stated that the position of the regulation introjected by approximation is between the identified regulation and the regulation introjected by avoidance, since it is more controlled and less autonomous than the identified regulation (Assor et al., 2009).

Furthermore, the more generic wording and lack of specific contextualization for competitive sport in the SMS-II (Pelletier et al., 1995) means that theoretically distinct constructs can be interpreted in a similar way, with larger overlap between the different dimensions. These issues do not seem to be relevant in the BRSQ, since it was designed to clearly reflect the differences between the different regulations in the continuum, ensuring greater discriminant validity (Lonsdale et al., 2008). However, it also faces challenges in distinguishing constructs that are closer in the continuum, as they are intrinsically similar (Lonsdale et al., 2008).

Thus, the most of discriminant validity problems in the SMS-II and the BRSQ can be justified by the conceptual proximity between the constructs, especially those that are closer on the continuum (Jelínek et al., 2021; Howard et al., 2017; Lonsdale et al., 2008). And that the wording of the items, the contextualization of the scales and individual differences contribute to these problems, especially in SMS-II. The cultural context where the scales are applied can also cause some of the discriminant validity problems, since the SDT was developed in a specific (Western) cultural context (Li et al., 2016). Therefore, if we consider a sporting context or a differentiated culture, the distinctions between motivational dimensions may not be as marked as predicted by the original model, which may lead to unexpected associations (Deci and Ryan, 1991).

In both scales, the factor related to introjected regulation was the one that, in most studies, presented items with standardized factor weights <0.5 (Cece et al., 2019; Chin et al., 2021; Jelínek et al., 2021; Nascimento et al., 2014; Pelletier et al., 2013; Pereira et al., 2024; Pineda-Espejel et al., 2016; Stenling et al., 2018; Vallejo-Reyes et al., 2018; Viciana et al., 2017; Viladrich et al., 2013). The introjected regulation factor, in both, presents items related to introjection by approach and by avoidance. In this sense, the items are not completely homogeneous, measuring different aspects of the main construct, leading to difficulty in converging the items into a single factor and, consequently, low standardized factor weights. Furthermore, the fact that there are items for approach and avoidance suggests that introjected regulation is not a unidimensional construct, but rather a multidimensional one (Howard et al., 2017). By remaining a multidimensional factor, it may hinder the interpretation of results, since an athlete motivated by approach introjection may benefit from different strategies than an athlete driven by introjection for avoidance. Following this line of thought, the distinction between two types of introjection would be a fundamental advance in terms of SDT.

In addition, some SMS-II validations (Chin et al., 2021; Jelínek et al., 2021; Li et al., 2016; Stenling et al., 2015; Vallejo-Reyes et al., 2018) reported standardized factor weights <0.5 in other regulations. In the identified regulation, the study developed by Vallejo-Reyes et al. (2018) justifies the value obtained by the overlap that the items of this factor have with the items of the integrated regulation, since both are associated with the athlete’s values and personal development. In the integrated regulation, Jelínek et al. (2021) obtained these low values in young people. According to Vallerand (1997), this regulation is not visible until reaching adulthood.

Furthermore, Lonsdale et al. (2008) states that the identified and integrated regulation factors are not clearly separable, and there may be an overlap between the items of these factors, as suggested by Vallejo-Reyes et al. (2018). Furthermore, values <0.5 were found in external regulation (Stenling et al., 2015) and amotivation (Chin et al., 2021; Li et al., 2016; Stenling et al., 2015).

The BRSQ presented several studies (Cece et al., 2019; Shokri et al., 2014; Monteiro et al., 2018; Moreno-Murcia et al., 2011; Tsitskari et al., 2015; Viladrich et al., 2013) with standardized factor weights <0.5: intrinsic motivation (Cece et al., 2019); integrated regulation (Shokri et al., 2014) and identified regulation (Cece et al., 2019; Monteiro et al., 2018; Moreno-Murcia et al., 2011; Tsitskari et al., 2015; Viladrich et al., 2013). Howard et al. (2017) concluded that intrinsic motivation and identified and integrated regulations are situated extremely close together on the self-determination continuum, which may create confusion between the items of these factors.

This confusion arises from the fact that these constructs correspond to a more autonomous motivation, and all involve a high degree of internalization, although identified and integrated regulations involve reasons external to behavior (Ryan and Deci, 2017). Furthermore, reinforcing their proximity is the “sense of self” (Howard et al., 2017), according to which, in integrated regulation, behavior is fully aligned with the “sense of self,” and in intrinsic motivation, behavior is already inherently autonomous and aligned with the self (Howard et al., 2017). The BRSQ, in turn, also presented reduced standardized factor weights in external regulation (Tsitskari et al., 2015; Viladrich et al., 2013) and in amotivation, only (Tsitskari et al., 2015).

### Model fit

Validation of the SMS-II was carried out in several contexts, most of which focused on testing the original model and verifying its structure and validity (Baaziz et al., 2023; Granero-Gallegos et al., 2018; Li et al., 2016; Pelletier et al., 2013, 2017; Pineda-Espejel et al., 2016; Rodrigues et al., 2021; Smohai et al., 2021; Viciana et al., 2017). Granero-Gallegos et al. (2018), although they obtained adequate adjustment values, used a sample of non-athlete students, compromising the validity of the observed data and not being able to generalize the results. Other studies, however, validated alternative models to the original, making some adjustments, such as error correlation, item elimination or factor combination (Barreira et al., 2022; Chin et al., 2021; Nascimento et al., 2014; Paic et al., 2017; Pereira et al., 2024; Pineda-Espejel et al., 2016; Viciana et al., 2017).

Regarding the BRSQ, several studies verified the fit with the original model, such as those by Alexe et al. (2022), Çetinkaya and Mutluer (2018), Filippos et al. (2019), Guedes et al. (2019), Hancox et al. (2015), Lonsdale et al. (2008), Monteiro et al. (2019) and Viladrich et al. (2011). Other studies, such as those by Cece et al. (2019), Francisco et al. (2019), Hancox et al. (2015), Luo et al. (2023), Monteiro et al. (2018), Moreno-Murcia et al. (2011) and Stenling et al. (2018) tested and validated alternative models, obtaining adequate fit.

Of the alternative models presented, only the models consisting of the factors: intrinsic motivation, autonomous extrinsic motivation, controlled extrinsic motivation and amotivation (Hancox et al., 2015); intrinsic motivation, integrated regulation, identified regulation, controlled extrinsic motivation and amotivation (Hancox et al., 2015); autonomous motivation, controlled motivation and amotivation (Alexe et al., 2022; Pineda-Espejel et al., 2016); two general motivation factors and six specific motivation factors (Luo et al., 2023); autonomous motivation, introjected regulation, external regulation and amotivation (Luo et al., 2023) presented adequate adjustment indices.

These results suggest that intrinsic motivation, integrated and identified regulation can be represented by autonomous motivation (Alexe et al., 2022; Luo et al., 2023) while introjected and external regulation can be represented by controlled motivation (Alexe et al., 2022; Hancox et al., 2015). The aggregation of these constructs is justified by their underlying characteristics. On the one hand, autonomous motivation reflects a high degree of internalization, in which behavior is perceived as an integral part of personal interests and values, in line with intrinsic motivation, integrated and identified regulation are. On the other hand, controlled motivation reflects a lower degree of internalization, in which behavior is performed in the face of external or internal pressures, in line with what external and introjected regulation are (Ryan and Deci, 2017).

### Invariance

The analysis of invariance between groups with different characteristics can contribute to expanding scientific knowledge and deepening the understanding of the universality of the factors underlying SDT (Deci and Ryan, 2008). By confirming the invariance between groups with different characteristics, the application of SDT is reinforced in different contexts, allowing a valid comparison between them. In this sense, the analysis of invariance is a fundamental step to confirm the universality of SDT and the scales that take it as a theoretical framework. In SDT, the existence of invariance is also particularly important when comparing motivational profiles in different cultures, ages or modalities.

The remaining validations that do not ensure invariance must be applied with caution, and there can be no generalization or comparison of the data. In these cases, it may also be necessary to adapt the scales to the population in question.

### Effect of correlations on the motivational continuum

According to the quasi-simplex pattern, the relationships should be more positive or negative the closer they are on the continuum (Ryan and Connell, 1989). However, the pattern that was found did not present data in accordance with the theory. While in the BRSQ only the correlations between the integrated-introjected and identified-introjected regulations do not correspond to the theoretical pattern. In SMS-II, the lack of agreement occurs in a larger number of correlations: intrinsic-introjected, integrated-introjected, integrated-external, identified-introjected and identified-external.

The controversy between the theoretical pattern and what happens, namely between the correlations integrated-introjected and identified-introjected regulation, can be explained through the duality of introjected regulation and its intermediate position on the continuum (Assor et al., 2009; Howard et al., 2017; Teixeira et al., 2022). This regulation implies partial internalization, in which behavioral regulation is not entirely external, but is also regulated by internal pressures (Ryan, 1982). Assor et al. (2009) suggested that this partial internalization can manifest itself in two ways: by approach or by avoidance. While introjection by approximation can support a positive correlation with the more autonomous forms, introjection by avoidance supports the positive correlation with the more controlled forms (Assor et al., 2009). Introjection functions as a transitional stage, incorporating both controlled and autonomous elements, explaining the different relationships with different types of regulation, namely with identified and integrated regulations that represent higher degrees of internalization. It is therefore possible that someone who values sport in their personal goals (integrated regulation) sometimes still feels an internal pressure to practice it (introjected regulation) (Deci and Ryan, 1991; Vallerand, 1997).

While these correlations appear in both scales, the positive correlation between intrinsic-introjected regulation appears only in SMS-II, which can be explained by several reasons. In part, due to the semantic formulation of SMS-II, which includes items that reflect both the search for pleasure (“it gives me pleasure to learn more about my sport”) and the search for self-esteem (“I feel better about myself when I do”), increasing the overlap between both correlations. But also due to the psychological overlap, since someone can feel such intrinsic pleasure when practicing sport (intrinsic motivation) but, at the same time, feel an internal pressure (introjected regulation) to continue practicing (Vallerand, 1997). Furthermore, the fact that this correlation is only positive in SMS-II may be due to the fact that the BRSQ was specifically designed for athletes (Clancy et al., 2017), emphasizing a clearer distinction between the constructs of intrinsic and introjected regulation.

Regarding the positivity of the correlations between integrated-external and identified-external regulations, it is important to understand that even during the internalization process, behavioral regulation continues to have an external aspect, since there may be an instrumental action, that is, carrying out the behavior to achieve personal goals (Deci and Ryan, 1991). However, it would be expected that these correlations would be negative, but if not, they would be low positive, since they represent different levels of self-determination (Vallerand, 1997).

The discrepancies can be conceptually justified by the dynamic and interdependent nature of human motivations: the continuum is a theoretical simplification that cannot fully capture the complexity of motivational processes in human beings; internalization, the coexistence of multiple forms of motivation and the conceptual overlap between dimensions explain why these correlations can arise, even when the model suggests clear distinctions (McLachlan et al., 2011).

All this highlighting the need for a careful analysis of the relationships between the different types of motivation and, possibly, a review of the operationalization of the constructs in the scale.

### Heterogeneity

The heterogeneity presented may reflect study variability (e.g., variety of participants), methodological variability (e.g., variety between study designs) or variability in sample characteristics (e.g., sex, age, sample size) (Bowden et al., 2011; Higgins and Green, 2008). In this sense, when collecting information from the selected studies, this sample variability was perceived. Taking this into account, the random effects model was chosen for the statistical analysis, since it takes this aspect into account, is more realistic and can be generalized to different populations.

## Limitations and future directions

This study has several limitations. First, the exclusion of validations published in languages other than English, Spanish, and Portuguese may introduce cultural bias and limit the diversity of the analysis. Additionally, translation variability across adaptations could affect scale interpretation. Second, incomplete data in some selected studies hindered a thorough evaluation, potentially impacting the consistency and generalizability of the findings. Furthermore, one of the limitations of this study is that some justifications (e.g., semantic overlap or translation issues) are speculated upon through the interpretation of various articles and discussions by various authors.

The future perspectives outlined by this study highlight the urgent need for a more in-depth and dynamic approach to understanding introjected regulation, an essential component of motivation that should not be treated as a fixed and homogeneous concept. To this end, it is imperative to develop assessment tools that can capture the subtleties of this process, avoiding simplifications that could compromise the validity of interpretations. In this sense, one of the central challenges identified is the need to reformulate the semantics of the items used in the measurement instruments, particularly in the SMS-II. Currently, some of the formulations may not accurately reflect the different nuances of more controlled motivations, leading to assessments that sometimes do not faithfully reflect the subjective experience of athletes. Also, it is important to rigorously testing measurement invariance across diverse cultures and sport levels to enhance cross-cultural validity and adopting more context-sensitive methodologies that account for athletes’ subjective experiences.

The future of research in this area therefore requires firm commitment to the evolution of theoretical and methodological models. Improving assessment instruments and adopting more dynamic and contextual perspectives will not only enrich scientific literature but will also provide more effective tools to optimize athlete motivation and development. Particularly crucial is the need to bridge the gap between psychometric measures and athletes’ lived experiences of motivation, ensuring assessments reflect the true complexity of motivational processes in sport contexts.

## Conclusion

The study showed that the BRSQ has greater internal consistency than the SMS-II, especially in the sub-scales of introjected and external regulation and amotivation. Although both scales face convergent and discriminant validity challenges, the SMS-II showed more inconsistencies, reflecting conceptual overlaps and cultural influences. Alternative models that group regulations into broader categories showed promise. Both scales showed invariance in different contexts, but generalization requires caution, highlighting the need for cultural adaptations. The correlations on the motivational continuum partially deviated from the quasi-simplex pattern of the SDT, reinforcing the dynamic complexity of motivation in sport.

To summarize, the BRSQ proved to be more robust, while the SMS-II requires more adjustments. Both are valid tools, but their use must consider contextual specificities.

## Data Availability

The original contributions presented in the study are included in the article/Supplementary material, further inquiries can be directed to the corresponding author.
