# Supplementary material for: Behavioral regulation in sport questionnaire and sport motivation scale-II: a scale comparison
Source: Front Psychol. 2025 Sep 11;16:1652580. doi: 10.3389/fpsyg.2025.1652580 (PMC12460324; doi:10.3389/fpsyg.2025.1652580)
Supplement: Supplementary file 1 [file Data_Sheet_1.pdf]

Tables summarizing the number of studies reporting reliability or validity issues by subscale

RELIABILITY	SMS-II	BRSQ
Intrinsic	3 of the 16 have values <0.70<br>· Barreira et al., 2022<br>· Granero-Gallegos et al.,2018<br>· Li et al., 2016	-x-
Integrated	2 of the 16 have values <0.70<br>· Jelínek et al., 2021<br>· Pelletier et al., 2017	1 of the 14 have values <0.70<br>· Shokri et al., 2014
Identified	1 of the 16 have values <0.70<br>· Viciana et al., 2017	3 of the 14 have values <0.70<br>· Viladrich et al., 2011<br>· Moreno-Murcia et al.,2011<br>· Monteiro et al., 2018
Introjected	9 of the 16 have values <0.70<br>· Barreira et al., 2022<br>· Chin et al., 2021<br>· Li et al., 2016<br>· Nascimento Junior et al., 2014<br>· Pereira et al.,2024<br>· Pineda-Espejel et al., 2016<br>· Smohai et al., 2021<br>· Vallejo-Reyes et al., 2018<br>· Viciana et al., 2017	-x-
External	5 of the 16 have values <0.70<br>· Barreira et al., 2022<br>· Chin et al., 2021<br>· Smohai et al., 2021<br>· Viciana et al., 2017<br>· Nascimento Junior et al. ,2014	2 of the 14 have values <0.70<br>· Viladrich et al., 2011<br>· Moreno-Murcia et al.,2011
Amotivation	7 of the 16 have values <0.70<br>· Barreira et al., 2022<br>· Chin et al., 2021<br>· Granero-Gallegos et al. ,2018<br>· Nascimento Junior et al. ,2014<br>· Pineda-Espejel et al., 2016<br>· Vallejo-Reyes et al., 2018<br>· Viciana et al., 2017	1 of the 14 have values <0.70<br>· Tsitskari et al., 2015

CONVERGENTE VALIDITY	*SMS-II*	*BRSQ*
Intrinsic	-X-	1 of the 16 shows a lack of convergent validity<br>· Cece et al., 2019
Integrated	-X-	1 of the 16 shows a lack of convergent validity<br>Shokri et al., 2014
Identified	1 of the 16 shows a lack of convergent validity<br>· Vallejo-Reyes et al., 2018	5 of the 16 shows a lack of convergent validity<br>· Cece et al., 2019<br>· Monteiro et al., 2018<br>· Moreno-Murcia et al., 2011<br>· Tsitskari et al., 2015<br>· Viladrich et al., 2013
Introjected	6 of the 16 shows a lack of convergent validity<br>· Nascimento et al. 2014<br>· Pineda-Espejel et al., 2016<br>· Viciana et al., 2017<br>· Vallejo-Reyes et al., 2018<br>· Jelínek et al. 2021<br>· Pereira et al., 2024	3 of the shows a lack of convergent validity<br>· Viladrich et al., 2013<br>· Cece et al., 2019<br>· Stenling et al., 2018
External	3 of the 16 shows a lack of convergent validity<br>· Stenling et al., 2015<br>· Chin et al., 2021<br>· Jelínek et al. 2021	2 of the 16 shows a lack of convergent validity<br>· Viladrich et al., 2013<br>· Tsitskari et al., 2015

Amotivation	3 of the 16 shows a lack of convergent validity<br>· Stenling et al., 2015<br>· Li et al., 2016<br>· Chin et al., 2021	1 of the 15 shows a lack of convergent validity<br>· Tsitskari et al., 2015

DISCRIMINANT VALIDITY	*SMS-II*	*BRSQ*
IM_INTE	7 of the 14<br>· Nascimento et al., 2014<br>· Stenling et al., 2015<br>· Viciana et al., 2017<br>· Granero-Gallegos et al., 2018<br>· Chin et al., 2021<br>· Jelínek et al., 2021<br>· Pereira et al., 2024	3 of the 13<br>· Monteiro et al., 2018<br>· Shokri et al., 2014<br>· Alexe et al., 2022
IM_IDENT	5 of the 14<br>· Nascimento Junior et al., 2014<br>· Viciana et al., 2017 SMSII6<br>· Granero-Gallegos et al., 2018<br>· Chin et al., 2021<br>· Jelínek et al., 2021 SMSII6	X
IM_INTRO	3 of the 14<br>· Nascimento Junior et al., 2014<br>· Viciana et al., 2017 SMSII6<br>· Granero-Gallegos et al., 2018	X
IM_EXT	X	X
IM_AM	X	1 of the 13<br>Stenling et al., 2018 T1
INTE_IDENT	7 of the 14<br>· Nascimento Junior et al., 2014<br>· Stenling et al., 2015<br>· Viciana et al., 2017 SMSII6/5<br>· Granero-Gallegos et al., 2018<br>· Li et al., 2016 stu1<br>· Jelínek et al., 2021 SMSII6<br>· Pereira et al., 2024	7 of the 13<br>· Lonsdale et al., 2008 stu2<br>· Moreno-Murcia et al., 2011<br>· Shokri et al., 2014<br>· Monteiro et al., 2018, 2019<br>· Alexe et al., 2022<br>· Luo et al., 2024 M1
INTE_INTRO	5 of the 14<br>· Nascimento Junior et al., 2014<br>· Viciana et al., 2017 SMSII6<br>· Granero-Gallegos et al., 2018<br>· Chin et al., 2021<br>· Jelínek et al., 2021 SMSII6	X
INTE_EXT	1 of the 14<br>· Granero-Gallegos et al., 2018	x
IDENT_INTRO	4 of the 14<br>· Nascimento Junior et al., 2014<br>· Viciana et al., 2017 SMSII6<br>· Granero-Gallegos et al., 2018<br>· Chin et al., 2021	x
INTRO_EXT	1 of the 14<br>· Stenling et al., 2015	7 of the 13<br>· Lonsdale et al., 2008 stu3<br>· Viladrich, et al., 2011, 2013<br>· Monteiro et al., 2018, 2019<br>· Stenling et al. 2018 T1<br>· Guedes et al. 2019 BRSQ6/8<br>· Alexe et al., 2022
INTRO_AM	X	1 of the 13<br>· Stenling et al., 2018 T1
EXT_AM	X	6 of the 13<br>· Viladrich, et al., 2011<br>· Moreno-Murcia et al. 2011<br>· Viladrich et al., 2013

		· Stenling et al., 2018 T2<br>· Guedes et al., 2019 BRSQ6/8<br>· Alexe et al., 2022

Standardized factor weights	SMS-II	BRSQ
Intrinsic	-X-	1 item <0.5 (Cece et al., 2019)
Integrated	1 item <0.5 (Jelínek et al., 2021)	1 item <0.5 (Shokri et al., 2014)
Identified	2 itens <0.5 (Vallejo-Reyes et al., 2018)	1 item <0.5 (Cece et al., 2019; Monteiro et al., 2018; Moreno-Murcia et al., 2011; Viladrich et al., 2013)<br>3 itens <0.5 (Tsitskari et al., 2015)
Introjected	Multiples itens <0.5 (Chin et al., 2021; Jelínek et al., 2021; Nascimento et al.,<br>2014; Pelletier et al., 2013; Pereira et al., 2024; Pineda-Espejel et al., 2016; Vallejo-Reyes et al., 2018; Viciana et al., 2017)	1 item <0.5 (Stenling et al., 2018; Viladrich et al., 2013),<br>2 itens <0.5 (Cece et al., 2019)
External	1 item <0.5 (Jelínek et al., 2021);	2 itens <0.5 (Viladrich et al., 2013)<br>All itens <0.5 (Tsitskari et al., 2015)
Amotivation	2 itens <0.5 (Chin et al., 2021; Li et al., 2016; Stenling et al., 2015)	All itens <0.5 (Tsitskari et al., 2015)

Tables relating to the adjustment and invariance indices for both scales

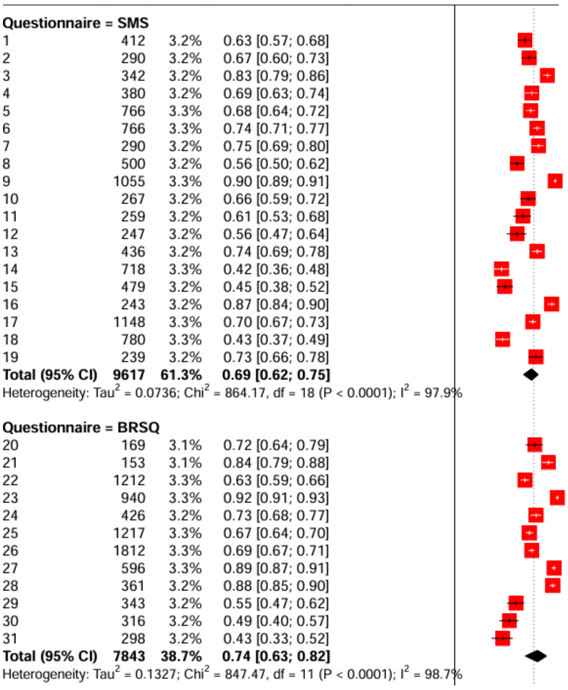

Figure 1

Effect of correlations between intrinsic motivation factors and integrated regulation

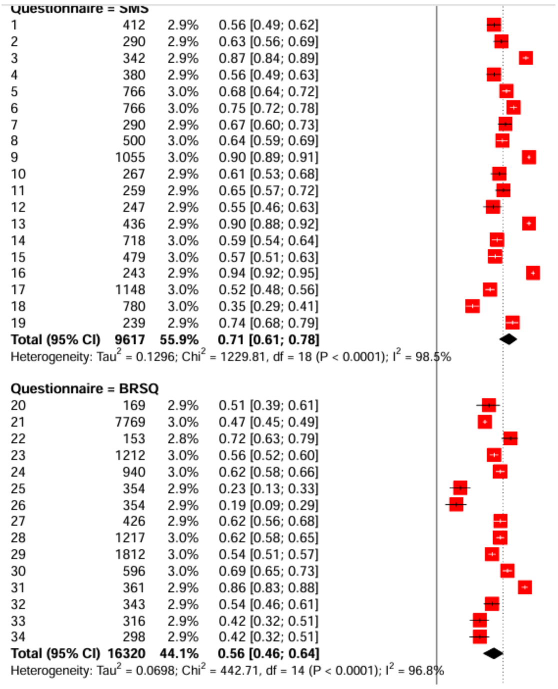

Figure 2

Effect of correlations between intrinsic motivation factors and identified regulation

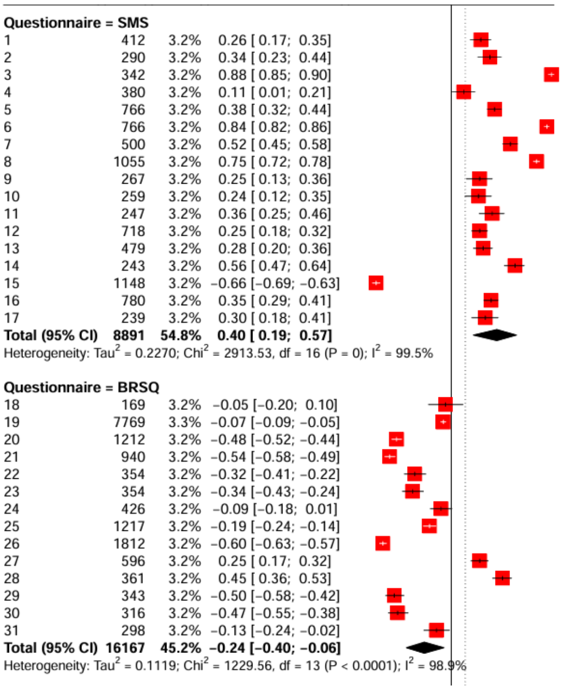

Figure 3

Effect of correlations between intrinsic motivation factors and introjected regulation

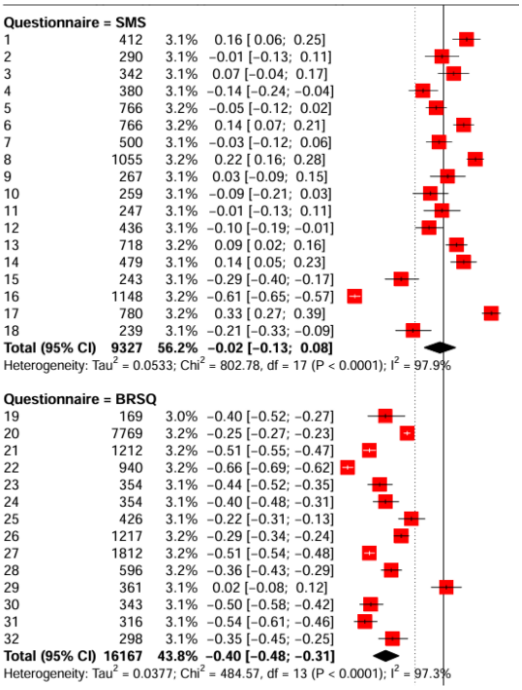

Figure 4

Effect of correlations between intrinsic motivation and external regulation factors

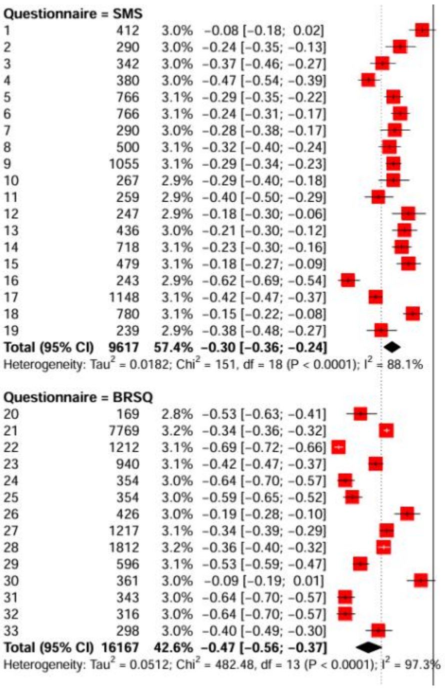

Figure 5

Effect of correlations between intrinsic motivation factors and amotivation regulation

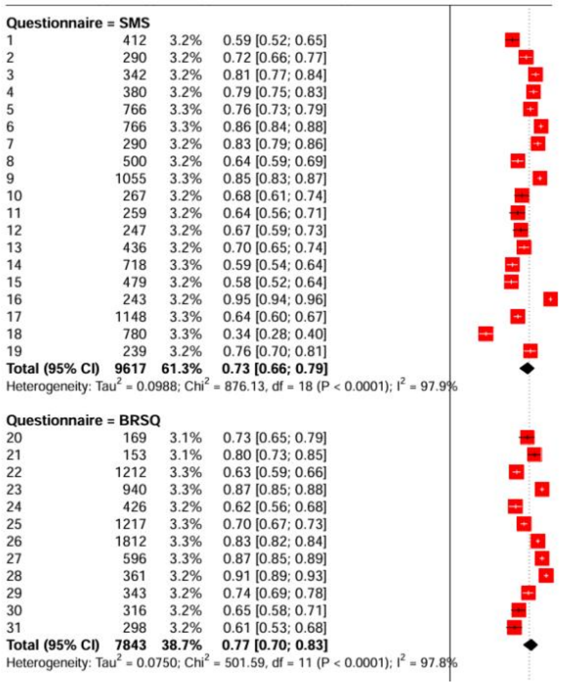

Figure 6

Effect of correlations between integrated regulation and identified regulation factors

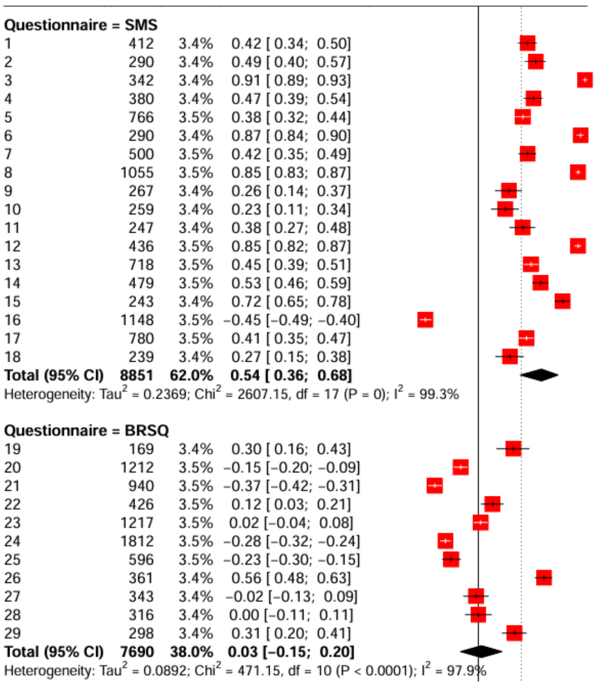

Figure 7

Effect of correlations between integrated regulation and introjected regulation factors

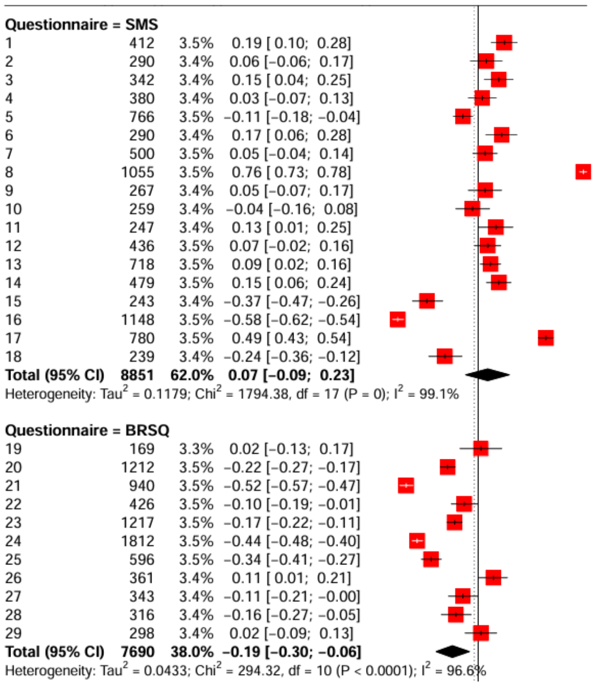

Figure 8

Effect of correlations between integrated regulation and external regulation factors

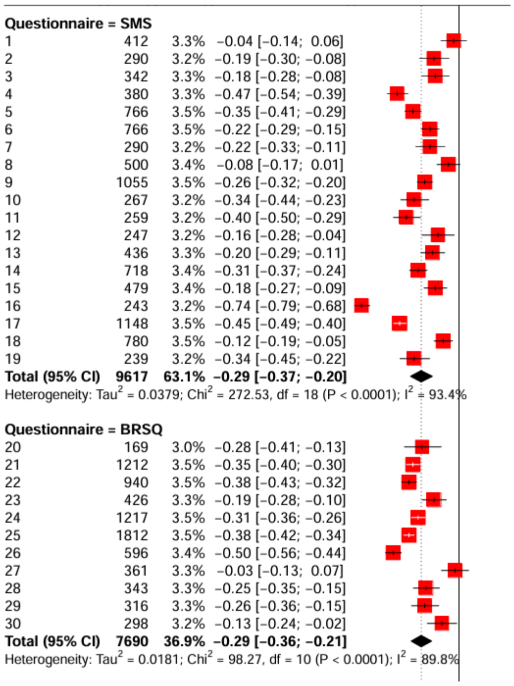

Figure 9

Effect of correlations between integrated regulation and amotivation factors

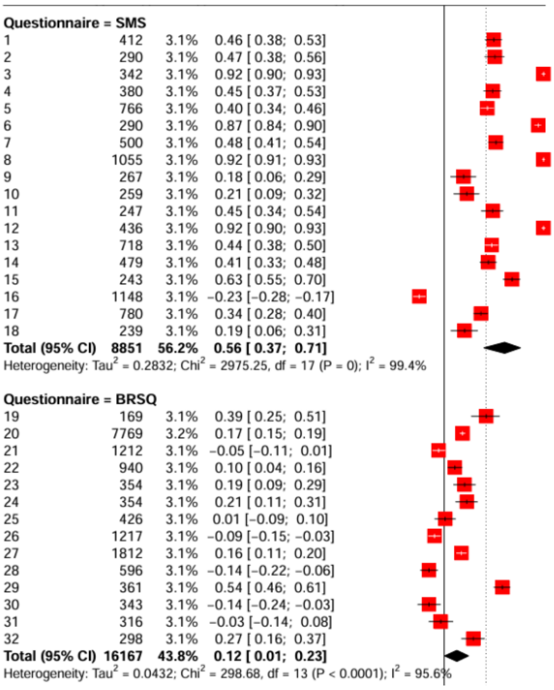

Figure 10

Effect of correlations between identified regulation factors and introjected regulation

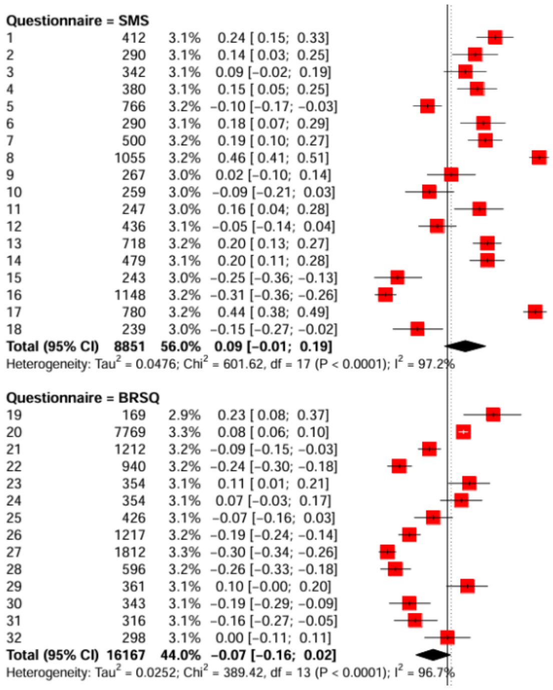

Figure 11

Effect of correlations between identified regulation factors and external regulation

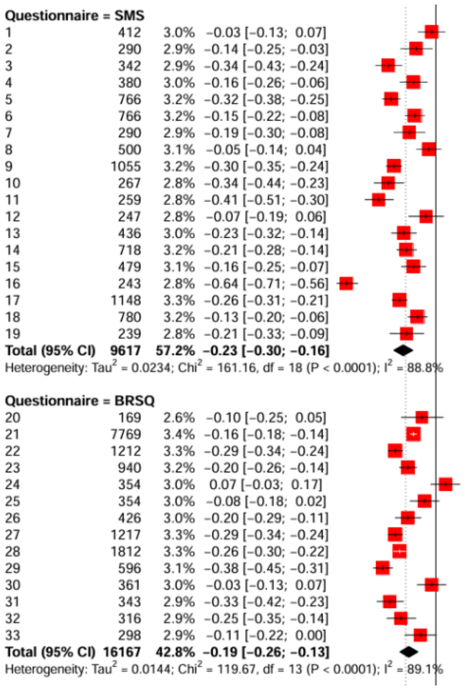

Figure 12

Effect of correlations between identified regulation factors and amotivation

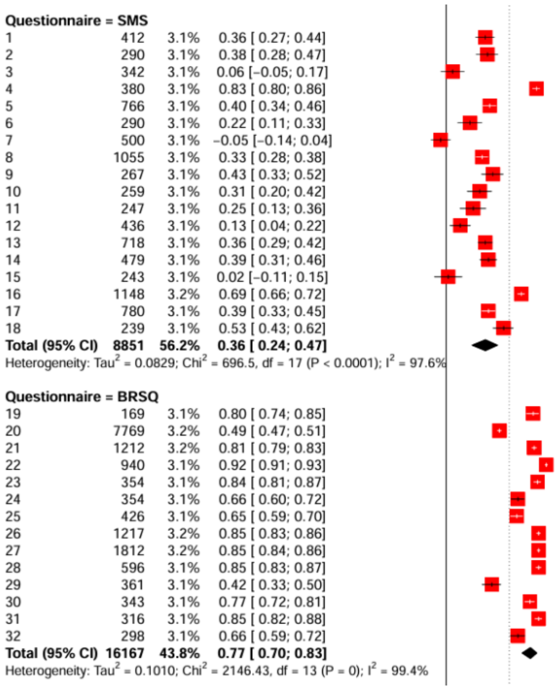

Figure 13

Effect of correlations between introjected regulation and external regulation factors

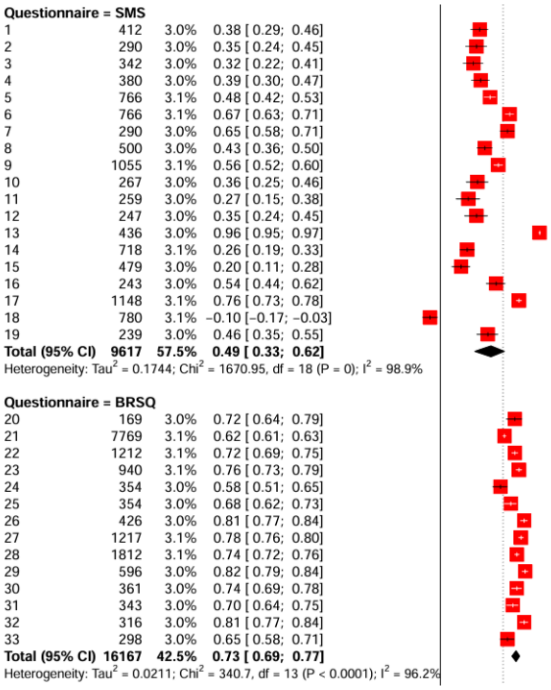

Figure 14

Effect of correlations between introjected regulation factors and amotivation

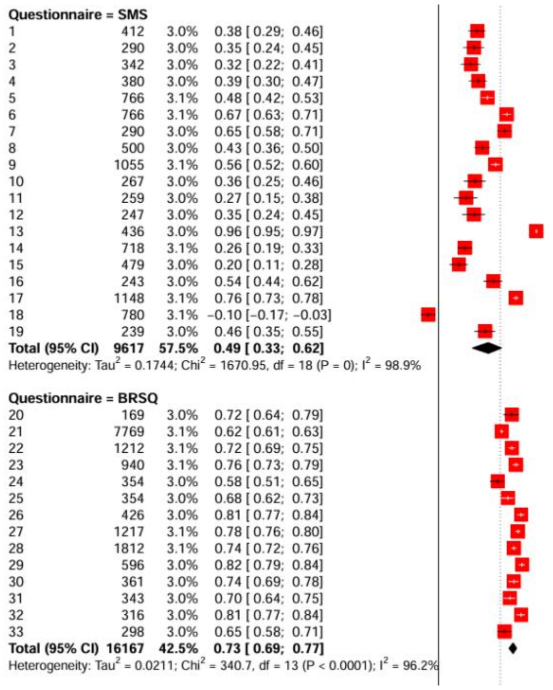

Figure 15

Effect of correlations between external regulation factors and amotivation
