# Supplementary material for: Behavioral regulation in sport questionnaire and sport motivation scale-II: a scale comparison
Source: Front Psychol. 2025 Sep 11;16:1652580. doi: 10.3389/fpsyg.2025.1652580 (PMC12460324; doi:10.3389/fpsyg.2025.1652580)
Supplement: Supplementary file 2 [file Supplementary_file_1.pdf]

Appendix A

Tables relating studies and characteristics of both scales

Table A.1

Studies and Characteristics of the SMS-II Sample

	Country	Sample	Sports level
Pelletier et al. (2013)	Canada	N=412 (218F; 104M; 90?); M=40.44 (SD = 13.66)	Adult athletes - Basketball, figure skating, hockey, soccer, running, swimming, etc.
		N= 290 (177F; 113M); M=17.41 (SD = 1.77)	Young athletes - provincial level basketball players (140) and swimmers (150)
Nascimento et al. (2014)	Brazil		
		N= 364 (151F; 213M) – 342 after excluded outliers; M=22.11 (SD = 4.86)	Athletes from the final phase of the Paraná Open Games 2013 - athletics, swimming, karate, cycling, tennis, chess, futsal, football, handball, basketball, rugby, volleyball and beach volleyball
		N=50; M=22.20 (SD = 4.43)	Futsal, swimming, athletics and judo
Stenling et al. (2015)	Sweden	N=380 (145F; 235M); M=17.2 (SD = 2.9)	Competitive athletes from a high school and sports teams
Pineda-Espejel et al. (2016)	Mexico	N=279 (131F; 143M); M=23.15 (SD=5.58)	High-performance athletes competing in the XXII Central American and Caribbean Games 2014 - Team sports (baseball, water polo, softball); Individual sports (boxing, fencing, artistic gymnastics, swimming, diving)
Viciana et al. (2017)	Spain	N=766 (263F;503M); M=13.71 (SD=1.30)	Teenage athletes - football, basketball, swimming, athletics, tennis, judo or volleyball
Paic et al. (2017)	Hungary	N=500 (181F; 319M); M=21.16 (SD = 6.45)	Sports teams and individual athletes
Granero-Gallegos et al. (2018)	Spain	N=1055 (536F; 519M); Female: M=13.93(SD =1.37); Male:13.87(SD = 1.42); Age between 12 and 17	High school students
		N=63 (32F; 31M); M=13.64 (SD = 1.23); Age between 12 and 17	High school students (non-athletes)
Li et al. (2016)	China	N=267 (67F; 197M; 3?); M=20.8 (SD=1.39)	College athletes (basketball, judo, karate and volleyball)
		N=259 (89F; 169M; 1?); M=20.5 (SD=1.38)	College athletes (boxing, football, gymnastics, tennis, taekwondo and wrestling)
		N=25 (17F; 8M); M=21.8 (SD=1.88)	College athletes (handball, karate, netball and triathlon)
Vallejo-Reyes et al. (2018)	Chile	N=221 (108F; 113M); M=22.0 (SD=3.29); Age between 18 and 38	Competition 132 - rugby, basketball, volleyball, hockey, table tennis, athletics, capoeira, handball, taekwondo and artistic gymnastics.<br>Recreation 89 - dance, volleyball, table tennis, swimming, climbing, dance, zumba, extreme cardio, yoga, Arabic dance, aero-box, weight training and basketball.
Pelletier et al. (2017)	France	N=247 (110F; 127M; 10?); M=17.07 (SD=2.80); Age between 13 and 25	Athletes - Handball, basketball, football, rugby, swimming, climbing, running, figure skating and cycling
		N=259 (170F; 89M); M=20.85 (SD=5.87); Age between 18 and 27	Athletes - Hockey, soccer, volleyball, American football, swimming, alpine skiing, cross-country running, rowing and other sports not mentioned
Chin et al. (2021)	Malaysia	N=436 (137F; 299M); M=16.44 (SD=1.22); Age between 14 and 21	Athletes
Smohai et al. (2021)	Hungary	N=1197 (598F; 599M); M=27.8 (SD = 10.3); Age between 11 and 67	Competition 718; recreation 479; team and individual sports
Jelinek et al. (2021)	Czech Republic	N=243 (123F; 120M); M=16.42 (SD = 1.31); Age between 14 and 19	Teenage athletes - Athletics, swimming, tennis, volleyball, basketball and football
Rodrigues et al. (2021)	Portugal	N=1148 (546F;602M); M=18.45 (SD=5.36)	Athletes - Team sports (basketball and football); individual sports (swimming and athletics)
Barreira et al. (2022)	Brazil	N=304 (126F; 178M); M=21.8 (SD=2.3); Age between 18 and 37	College students
		N=441 (263F;178M); M=21.8 (SD=3.9); Age between 18 and 35	College students
		N=310 (140F; 170M); M=21.9 (SD=2.7)	College students
M. Baaziz et al. (2023)	Tunisia	N=780 (348F; 432M); M=18.97 (SD= 4.01); Age between 13 and 30	Athletes - Team sports (football, handball, volleyball and basketball) and 388 athletes; Individual sports (kick boxing, English boxing, Taekwondo, athletics, gymnastics and swimming)
Pereira et al. (2024)	Portugal	N=239 (107F; 132M); M=14.39 (SD=1.35); Age between 12 and 17	Teenage athletes - Handball, athletics, basketball, football, swimming and volleyball

Table A.2

Studies and Characteristics of the BRSQ Sample

	Country	Sample	Sports level
Lonsdale et al. (2008)	New Zealand	N=382 (176F; 206M); M=25.9; ages 18-58	New Zealand Sports Academy; 20 different sports
		N=343 (183F; 160M) M=24.47; ages 14-57	New Zealand Sports Academy; 23 different sports
		N=316 (173F; 141M; 2?); M=19.4; ages 17-43	Non-elite students at a New Zealand university; 38 different sports
		N=34; M=22.36	Rugby three players from teams competing at an amateur club in New Zealand
Viladrich et al. (2011)	Spain	N=169 (29F; 140M); M=20.65 (SD=2.93)	Athletes - individual (gymnastics, swimming and badminton) or team (basketball, futsal, volleyball, handball and hockey)
Moreno-Murcia et al. (2011)	Spain	N=298 (79F; 219M); M=14 (SD=1.79)	Athletes - football, basketball, volleyball, handball, athletics, rhythmic gymnastics, canoeing, swimming, tennis, judo, karate, table tennis, table tennis and futsal
Viladrich et al. (2013)	France Greece Norway Spain England	N=7769 (13%F; 87%M); M=11.76 (SD=1.42); ages 9-15	Football players
Shokri et al. (2014)	Spain	N=153 (9F; 144M); M=28.65 (SD = 8.84); ages 15 to 52	Football, basketball, handball and skiing coaches
Hancox et al. (2015)	United Kingdom	N=1212 (948F; 259M; 5?); M=20.19 (SD=10.41); ages 10-79; teens (10-17) = 572; adults (18-79) = 636	Recreational dancers (n = 344); professional dancers (n = 868)
Tsitskari et al. (2015)	Greece	N=158 (37F; 121M); ages 10-13	Volleyball, football and basketball sports clubs
Çetinkaya and Mutluer (2018)	Türkiye	N=681 (286F; 395M); 9 (51.2%) of participants are between 18 and 21 years old; 303 (44.5%) of participants are between 22 and 25 years old; 22 (3.2%) of participants are between 26 and 29 years old; 7 (1%) of participants are >30 years old	Student-athletes who are licensed athletes and are still studying
Monteiro et al. (2018)	Portugal	N=940 (232F; 708M); M=17.63 (SD=4.32); ages 13-36	Athletes - football, basketball, athletics, swimming
Stenling et al. (2018)	Sweden	N=354 (48%F; 52%M); M=17.2 (SD=1.16); ages 15-21	Young competitive athletes (skiers=46%; floorball players=54%)
Francisco et al. (2019)	Spain	N=426 (49.3%F; 50.7%M); M=17.89 (SD = 3.58)	Individual or team sports
Cece et al. (2019)	France	T1= 657; T2=554; T3=435 (263F; 473M); M=16.4 (SD=1.71)	Young athletes - basketball (188), football (182), rugby (127), handball (97), judo (43), dance (30), athletics (26), swimming (16), boxing (5), cycling (4), golf (3), gymnastics (n = 3), fencing (n = 2), weightlifting (n = 2), karate (n = 2), hockey (1), football (1), skiing (1), tennis (1), volleyball (1) and wakeboarding (1).
Guedes et al. (2019)	Brazil	N=1217 (410F; 807M); ages 12-17	Young athletes participating in the Paraná Youth Games in 2015 - basketball, handball, volleyball, futsal, football, athletics, swimming, cycling, gymnastics, judo, karate, taekwondo and tennis
Filippos et al. (2019)	Greece	N=390 (77.2%F; 22.8%M); 15-18 - 37.4%; 19-29 - 23.8%; 30-39 - 18.5%; 40-49 - 14.4%; >50 - 5.9%	Traditional dances (249); Modern dance (141)
Monteiro et al. (2019)	Portugal	N=1812 (582F; 596M); M=17.72 (SD=5.51); ages 15 to 59	Football, basketball, swimming and water sports
Alexe et al. (2022)	Romania	N=596(272F; 324M); M=22.91 (SD=5.84); ages 18-52	Professional athletes - 273 individuals (athletics, gymnastics, rowing, weightlifting, cycling or Olympic shooting) and 323 sports (football, basketball, volleyball, rugby, ice hockey or handball)
Luo et al. (2023)	China	N=361 (92F; 269M); M=20.45 (SD = 1.41)	College athletes - 232 professionals; 129 competitive athletes

Appendix B

Tables relating to standardized factor weights, reliability values, average variance extracted values and discriminant validity of both scales

Table B.3

Standardized factor weights, reliability values, average variance extracted values and discriminant validity of SMS-II

	Pelletier et al. (2013)		Nascimento et al. (2014)	Stenling et al. (2015)	Pineda-Espejel et al. (2016)	Viciana et al. (2017)		Granero-Gallegos et al. (2018)	Paic et al. (2017)		Li et al. (2016)		Vallejo-Reyes et al. (2018)	Pelletier et al. (2017)	Chin et al. (2021)	Smohai et al. (2021)	Jelinek et al. (2021)		Rodrigues et al. (2021)					Barreira et al. (2022)	Baaziz et al. (2023)	Pereira et al. (2024)
	Study1	Study2				SMSII6	SMSII5		M1	M2	Study1	Study2				IN	6factors	4factors	Collective	Individual	Feminine	Masculine	Total sample			
Intrinsic	α = 0.88 AVE=0.71	IN AVE=0.72	α=0.71 CC=0.71 AVE=0.47	IN AVE=0.61	α=0.76 AVE=0.63	α=0.74 ω=0.74 AVE =0.49	α=0.74 ω=0.74 AVE =0.49	α=0.68 ω=0.89 AVE=0.38	α=0.89 CR=0.89 AVE =0.67	α=0.89 CR=0.94 AVE =0.89	α=0.75 CR=0.75 AVE =0.50	α=0.69 CR=0.69 AVE =0.44	α=0.70 AVE=0.50	α=0.77 AVE=0.55	α=0.72 AVE=0.46	α=0.80 IN	CR=0.82 AVE=0.61	IN IN	CR=0.83 IN	CR=0.86 IN	CR=0.84 IN	CR=0.85 IN	AVE=0.66	1. 0.77<br>2. 0.63<br>3. 0.63<br>IN	α=0.95 ω=0.95 AVE=0.86	CR=0.77 AVE=0.54
17.	0.80	0.85	0.78	0.71	0.85	0.77	0.75	0.68			0.75	0.75	0.53	0.62	0.60		0.71	0.70	0.63	0.66	0.61	0.64			0.92	0.61
9.	0.86	0.85	0.60	0.75	0.79	0.68	0.70	0.61			0.69	0.67	0.76	0.86	0.67		0.75	0.71	0.88	0.88	0.87	0.90			0.94	0.65
3.	0.86	0.77	0.59	0.87	0.73	0.65	0.65	0.54			0.68	0.55	0.80	0.73	0.75		0.88	0.83	0.86	0.89	0.89	0.88			0.93	0.71
Integrated	α = 0.80 AVE=0.57	IN AVE=0.58	α=0.70 CC=0.73 AVE=0.49	IN AVE=0.44	α=0.73 AVE=0.59	α=0.74 ω=0.75 AVE =0.50	α=0.74 ω=0.74 AVE =0.49	α=0.72 ω=0.87 AVE=0.46	α=0.85 CR=0.83 AVE =0.70	α=0.85 CR=0.85 AVE =0.74	α=0.70 CR=0.71 AVE =0.46	α=0.70 CR=0.70 AVE =0.43	α=0.84 AVE=0.66	α=0.67 AVE=0.51	α=0.73 AVE=0.57	α=0.76 IN	CR=0.68 AVE=0.42	IN IN	CR=0.77 IN	CR=0.76 IN	CR=0.76 IN	CR=0.77 IN	AVE=0.56	1. 0.79<br>2. 0.80<br>3. 0.78<br>IN	α=0.94 ω=0.94 AVE=0.84	CR=0.74 AVE=0.50
4.	0.78	0.68	0.69	0.66	0.72	0.68	0.69	0.68	0.86	0.86	0.75	0.64	0.79	0.62	0.78		0.50	0.49	0.62	0.65	0.61	0.62			0.90	0.67
11.	0.73	0.77	0.69	0.59	0.86	0.73	0.72	0.73	0.82	0.82	0.61	0.61	0.84	0.65	0.72		0.75	0.70	0.85	0.80	0.82	0.84			0.93	0.70
14.	0.75	0.79	0.67	0.73	0.71	0.70	0.69	0.63			0.66	0.72	0.81	0.62	0.77		0.67	0.68	0.71	0.69	0.72	0.71			0.92	0.79
Identified	α = 0.82 AVE=0.64	IN AVE=0.67	α=0.77 CC=0.77 AVE=0.54	IN AVE=0.60	α=0.74 AVE=0.61	α=0.69 ω=0.70 AVE =0.43	α=0.70 ω=0.73 AVE =0.47	α=0.76 ω=0.85 AVE=0.52	α=0.85 CR=0.87 AVE =0.69	α=0.85 CR=0.85 AVE =0.65	α=0.70 CR=0.71 AVE =0.45	α=0.74 CR=0.74 AVE =0.48	α=0.71 AVE=0.20	α=0.81 AVE=0.59	α=0.75 AVE=0.51	α=0.80 IN	CR=0.82 AVE=0.60	IN IN	CR=0.70 IN	CR=0.74 IN	CR=0.71 IN	CR=0.72 IN	AVE=0.54	1. 0.76<br>2. 0.77<br>3. 0.83<br>IN	α=0.87 ω=0.87 AVE=0.73	CR=0.82 AVE=0.62
12.	0.78	0.90	0.71	0.77	0.85	0.70	0.75	0.70	0.75	0.75	0.75	0.81	0.75	0.74	0.67		0.73	0.72	0.69	0.65	0.64	0.67			0.88	0.71
6.	0.70	0.75	0.62	0.79	0.74	0.58	0.64	0.70	0.83	0.83	0.67	0.63	0.20	0.78	0.72		0.80	0.79	0.63	0.69	0.62	0.65			0.81	0.83
18.	0.91	0.63	0.82	0.76	0.74	0.69	0.67	0.76	0.91	0.90	0.59	0.63	0.11	0.78	0.75		0.80	0.79	0.72	0.75	0.75	0.72			0.88	0.73
Introjected	α = 0.70 AVE=0.40	IN AVE=0.41	α=0.61 CC=0.56 AVE=0.35	IN AVE=0.43	α=0.51 AVE=0.41	α=0.64 ω=0.66 AVE =0.40	IN	α=0.78 ω=0.86 AVE=0.38	α=0.79 CR=0.78 AVE =0.64	α=0.79 CR=0.80 AVE =0.68	α=0.73 CR=0.73 AVE =0.48	α=0.69 CR=0.69 AVE =0.43	α=0.39 AVE=0.27	α=0.71 AVE=0.50	α=0.46 AVE=0.21	α=0.68 IN	CR=0.71 AVE=0.46	CR=0.71 AVE=0.46	CR=0.80 IN	CR=0.82 IN	CR=0.81 IN	CR=0.82 IN	AVE=0.60	1. 0.39<br>2. 0.33<br>3. 0.32<br>IN	α=0.92 ω=0.92 AVE=0.81	CR=0.63 AVE=0.36
1.	0.59	0.71	0.47	0.70	0.44	0.40		0.54	0.68	0.68	0.55	0.58	0.34	0.63	0.24		0.73	0.74	0.69	0.71	0.72	0.74			0.86	0.66
16.	0.62	0.67	0.72	0.59	IN	0.67		0.73	0.91	0.91	0.82	0.69	0.06	0.63	0.67		0.78	0.78	0.82	0.86	0.80	0.82			0.93	0.56
7.	0.68	0.47	0.38	0.68	0.79	0.54		0.56			0.68	0.69	0.83	0.76	0.34		0.48	0.46	0.74	0.76	0.72	0.76			0.91	0.43
External	α = 0.74 AVE=0.61	IN AVE=0.52	α=0.69 CC=0.71 AVE=0.47	IN AVE=0.38	α=0.75 AVE=0.62	α=0.54 ω=0.55	α=0.65 ω=0.66	α=0.72 ω=0.86 AVE=0.44	α=0.85 CR=0.86	α=0.86 CR=0.86	α=0.76 CR=0.78	α=0.76 CR=0.77	α=0.69 AVE=0.53	α=0.83 AVE=0.65	α=0.62 AVE=0.45	α=0.62 IN	CR=0.72 AVE=0.48	CR=0.72 AVE=0.48	CR=0.90 IN	CR=0.89 IN	CR=0.91 IN	CR=0.89 IN	AVE=0.77	1. 0.74<br>2. 0.68<br>3. 0.69<br>IN	α=0.93 ω=0.93 AVE=0.82	CR=0.81 AVE=0.59

						AVE =0.30	AVE =0.49		AVE =0.67	AVE =0.66	AVE =0.55	AVE =0.53														
5.	0.94	0.59	0.75	0.67	0.78	0.65	0.64	0.72	0.85	0.85	0.84	0.76	0.81	0.85	0.62		0.80	0.83	0.89	0.87	0.88	0.90			0.91	0.65
8.	0.79	0.76	0.71	0.71	0.95	0.70	0.76	0.67	0.74	0.74	0.80	0.82	0.67	0.80	0.61		0.82	0.79	0.78	0.77	0.77	0.74			0.92	0.68
15.	0.57	0.74	0.52	0.44	0.58	0.53	X	0.60	0.86	0.86	0.55	0.59	0.70	0.76	0.77		0.38	0.38	0.93	0.90	0.91	0.90			0.89	0.77
Amotivation	α = 0.81<br>AVE= 0.56	IN<br>AVE=0.60	α=0.78<br>CC=0.69<br>AVE=0.44	IN<br>AVE=0.44	α=0.68<br>AVE=0.79	α=0.64<br>ω=0.65<br>AVE =0.38	α=0.64<br>ω=0.64<br>AVE =0.44	α=0.64<br>ω=0.87<br>AVE=0.37	α=0.80<br>CR=0.81<br>AVE =0.59	α=0.80<br>CR=0.85<br>AVE =0.65	α=0.70<br>CR=0.72<br>AVE =0.47	α=0.73<br>CR=0.73<br>AVE =0.48	α=0.76<br>AVE=0.61	α=0.77<br>AVE=0.54	α=0.53<br>AVE=0.22	α=0.74<br>IN	CR=0.81<br>AVE=0.58	CR=0.81<br>AVE=0.59	CR=0.86<br>IN	CR=0.84<br>IN	CR=0.85<br>IN	CR=0.86<br>IN	AVE=0.69	1. 0.73<br>2. 0.65<br>3. 0.61<br>IN	α=0.95<br>ω=0.95<br>AVE=0.90	CR=0.78<br>AVE=0.57
2.	0.74	0.95	0.67	0.87	0.70	0.56	0.57	0.53	0.84	0.84	0.47	0.59	0.68	0.83	0.51		0.86	0.87	0.79	0.78	0.78	0.77			0.95	0.81
10.	0.76	0.77	0.56	0.73	0.96	0.59	0.59	0.61	0.65	0.65	0.76	0.74	0.85	0.77	0.56		0.71	0.73	0.84	0.83	0.83	0.82			0.95	0.71
13.	0.74	0.77	0.70	0.18	0.98	0.69	0.66	0.68	0.80	0.80	0.79	0.73	0.80	0.60	0.30		0.71	0.68	0.82	0.82	0.82	0.85			0.94	0.68
Intrinsic effective									x	0.98																
44									0.77	0.83																
45									0.77	0.75																
46									0.78	0.84																
Intrinsic cognitive									X	0.90																
30									0.79	0.84																
13									0.69	0.70																
21									0.72	0.74																
Autonomous					In model 2 α=0.88													CR=0.90<br>AVE=0.51								
Controlled					In model 2 α= 0.75																					
Discriminant validity	-x-	-x-	Problems in:<br>Im/integ;<br>Im/ident;<br>Im/intro;<br>Integ/ident;<br>Integ/intro;<br>ID/intro	Problems in:<br>Im/integ;<br>Integ/ident;<br>Intro/ext	-x-	Problems in:<br>Im/integ;<br>Im/ident;<br>Im/intro;<br>Integ/ident;<br>Integ/intro;<br>Ident/intro	Problems in:<br>Im/integ;<br>Integ/intro	Problems in:<br>Im/integ;<br>Im/ident;<br>Im/intro;<br>Integ/ident;<br>Integ/intro;<br>Integ/ext;<br>Ident/intro	-x-	-x-	Problems in:<br>Inte/ident	-x-	No data	-x-	Problems in:<br>Im/integ;<br>Im/ident;<br>Integ/intro;<br>Ident;intro	No data	Problems in:<br>Im/integ;<br>Im/ident;<br>Integ/ident;<br>Integ/intro	-x-					-x-	No data	-x-	Problems in:<br>Im/integ;<br>Ident/integer

-x no issues report
*Obtained from Jelínek et al. (2021)

Table B.4

Standardized factor weights, reliability values, average variance extracted values and discriminant validity of the BRSQ

	Lonsdale et al. (2008)			Viladrich et al. (2011)	Moreno-Murcia et al. (2011)	Viladrich et al. (2013)	Shokri et al. (2014)	Hancox et al. (2015)	Tsitskari et al. (2015)	Çetinkaya, T. and C. Mutluer (2018)	Monteiro et al. (2018)	Stenling et al. (2018)		Francisco et al. (2018)	Cece et al. (2019)			Guedes et al. (2019)		Filippos et al. (2019)	Monteiro et al. (2019)	Alexe et al. 2022)	Luo et al. (2024)	
	Study1	Study2	Study3									T1	T2		T1	T2	T3	BRSQ 6	BRSQ 8				M1	Mfinal
General IM	IN	α = 0.92 AVE=0.75	α = 0.85 AVE=0.61	α = 0.80 AVE=0.67	α = 0.75	IN AVE=0.50	α = 0.76 AVE=0.57	IN AVE=0.66	α = 0.85 AVE=0.50	α = 0.86 AVE=0.55	α = 0.82 CR=0.82 AVE=0.54	ω= 0.80 AVE= 0.55	ω= 0.83 AVE=0.58	α = 0.78 AVE=0.66	IN AVE= 0.36	IN AVE= 0.39	IN AVE= 0.42	IN AVE=0.60	α = 0.82 IN	α = 0.84 IN	CR=0.85 AVE=0.60	α =0.85 ω=0.87 AVE=0.52	ω=0.96 AVE= 0.84	IN AVE= 0.64
1.	IN	0.81	0.81	0.86	IN	0.62	0.78	0.86	0.66	0.71	0.81	0.71	0.74	0.80	0.51	0.60	0.71	0.73	IN		0.86	0.85	0.93	
11.	IN	0.94	0.85	0.90	IN	0.53	0.82	0.90	0.85	0.77	0.78	0.73	0.75		0.46	0.54	0.55	0.81	IN		0.84	0.83	0.94	
16.	IN	0.88	0.82	0.77	IN	0.92	0.70	0.76	0.68	0.75	0.64	0.88	0.89	0.83	0.75	0.78	0.65	0.78	IN		0.68	0.53	0.86	0.80
19.	IN	0.83	0.63	0.71	IN	0.69	0.75	0.72	0.62	0.74	0.67	0.62	0.65		0.64	0.55	0.66	0.77	IN		0.74	0.63	0.94	
Integrated	α = 0.71 AVE=0.46	α = 0.79 AVE=0.50	α = 0.76 AVE=0.49	α = 0.75 AVE=0.53	α = 0.78 AVE=0.43	IN IN	α = 0.67 AVE=0.39	IN AVE=0.58	IN AVE=0.68	α = 0.84 AVE=0.57	α = 0.75 CR=0.74 AVE=0.44	IN IN	IN IN	α = 0.83 AVE=0.71	IN IN	IN IN	IN IN	IN AVE=0.56	α = 0.71 AVE=0.56	α = 0.92 IN	CR=0.80 AVE=0.51	α =0.82 ω=0.90 AVE=0.54	ω=0.94 AVE= 0.79	IN AVE= 0.73
2.	0.64	0.63	0.73	0.79	0.62	IN	0.71	0.80	0.85	0.78	0.73			0.85				0.71	0.71		0.67	0.75	0.83	0.81
3.	0.65	0.66	0.77	0.76	0.64	IN	0.54	0.78	0.87	0.82	0.60			0.83				0.78	0.78		0.77	0.75	0.90	
8.	0.74	0.79	0.70	0.71	0.68	IN	0.47	0.81	0.86	0.74	0.59							0.79	0.79		0.73	0.75	0.92	
24.	X	0.74	0.59	0.65	0.68	IN	0.74	0.63	0.62	0.66	0.67							0.71	0.71		0.71	0.69	0.91	0.90
Identified	α = 0.73 AVE=0.46	α = 0.82 AVE=0.54	α = 0.77 AVE=0.48	α = 0.68 AVE=0.45	α = 0.68 AVE=0.33	IN AVE=0.34	α = 0.79 AVE=0.56	IN AVE=0.49	IN AVE=0.30	α = 0.86 AVE=0.73	α = 0.70 CR=0.69 AVE=0.40	ω= 0.72 AVE= 0.47	ω= 0.74 AVE=0.49	α = 0.72 AVE=0.52	IN AVE= 0.34	IN AVE= 0.30	IN AVE= 0.29	IN AVE=0.57	α = 0.74 AVE=0.57	α = 0.76 IN	CR=0.71 AVE=0.38	α =0.75 ω=0.84 AVE=0.50	ω=0.94 AVE= 0.80	IN AVE= 0.62
9.	0.77	0.74	0.80	0.57	0.49	0.59	0.75	0.77	0.98	0.83	0.60	0.68	0.69	0.70	0.57	0.54	0.44	0.75	0.75		0.54	0.61	0.83	0.79
17.	0.73	0.73	0.57	0.70	0.61	0.64	0.62	0.53	0.32	0.80	0.49	0.61	0.62		0.63	0.51	0.56	0.77	0.77		0.69	0.64	0.90	
20.	0.60	0.67	0.82	0.68	0.52	0.64	0.79	0.85	0.32	0.80	0.64	0.67	0.69	0.74	0.48	0.45	0.58	0.76	0.76		0.55	0.68	0.95	
22.	0.58	0.79	0.53	0.71	0.65	0.44	0.81	0.60	0.19	0.97	0.69	0.77	0.80		0.64	0.68	0.55	0.75	0.75		0.67	0.70	0.90	
Introjected	α = 0.87 AVE=0.70	α = 0.88 AVE=0.67	α = 0.91 AVE=0.72	α = 0.73 AVE=0.55	α = 0.77 AVE=0.42	IN AVE=0.34	IN AVE=0.53	IN AVE=0.66	α = 0.86 AVE=0.71	α = 0.74 AVE=0.52	α = 0.83 CR=0.82 AVE=0.54	ω= 0.74 AVE= 0.41	ω= 0.77 AVE=0.43	α = 0.72 AVE=0.58	IN AVE= 0.29	IN AVE= 0.27	IN AVE= 0.35	IN AVE=0.64	α = 0.83 AVE=0.64	α = 0.88 IN	CR=0.86 AVE=0.61	α =0.78 ω=0.88 AVE=0.51	ω=0.93 AVE= 0.76	ω=0.93 AVE= 0.76
4.	0.82	0.86	0.84	0.78	0.62	0.74	0.67	0.80	0.89	0.80	0.78	0.69	0.72	0.76	0.85	0.62	0.95	0.73	0.73		0.77	0.68	0.87	0.87
6.	X	0.78	0.88	0.79	0.65	0.57	0.68	0.84	0.86	0.72	0.70	0.43	0.44		0.37	0.46	0.35	0.83	0.83		0.85	0.68	0.90	0.90
12.	0.81	0.72	0.78	0.64	0.63	0.13	0.82	0.80	0.81	0.65	0.76	0.74	0.76		0.20	0.28	0.31	0.68	0.68		0.80	0.72	0.79	0.79
18.	0.87	0.90	0.88	0.75	0.68	0.68	0.74	0.81	0.81	0.71	0.70	0.65	0.67	0.76	0.51	0.65	0.51	0.75	0.75		0.78	0.64	0.93	0.93
External	α = 0.85 AVE=0.68	α = 0.93 AVE=0.77	α = 0.91 AVE=0.71	α = 0.61 AVE=0.49	α = 0.63 AVE=0.48	IN AVE=0.20	IN AVE=0.58	IN AVE=0.74	α = 0.77 AVE=0.00	α = 0.79 AVE=0.54	α = 0.90 CR=0.90 AVE=0.71	ω= 0.74 AVE= 0.41	ω= 0.81 AVE=0.52	α = 0.77 AVE=0.65	IN AVE= 0.39	IN AVE= 0.37	IN AVE= 0.46	IN AVE=0.58	α = 0.84 AVE=0.58	α = 0.86 IN	CR=0.91 AVE=0.73	α =0.85 ω=0.90 AVE=0.62	ω=0.94 AVE= 0.80	ω=0.94 AVE= 0.80
10.	0.78	0.86	0.82	0.68	0.64	0.50	0.62	0.85	-0.007	0.70	0.85	0.52	0.61		0.53	0.64	0.61	0.72	0.72		0.86	0.79	0.92	0.92
14.	0.88	0.89	0.85	0.83	0.73	0.51	0.89	0.92	0.02	0.83	0.91	0.59	0.67	0.79	0.67	0.56	0.79	0.77	0.77		0.79	0.85	0.87	0.87
15.	0.83	0.88	0.84	0.61	0.74	0.39	0.90	0.81	0.02	0.83	0.85	0.68	0.76		0.67	0.79	0.63	0.78	0.78		0.91	0.85	0.89	0.89
23.	0.81	0.88	0.85	0.66	0.65	0.36	0.57	0.77	0.01	0.55	0.75	0.75	0.83	0.82	0.61	0.72	0.66	0.77	0.77		0.89	0.67	0.89	0.89
Amotivation	α = 0.87 AVE=0.65	α = 0.90 AVE=0.69	α = 0.91 AVE=0.71	α = 0.71 AVE=0.58	α = 0.83 AVE=0.55	IN AVE=0.57	IN AVE=0.59	IN AVE=0.71	α = 0.66 AVE=0.00	α = 0.80 AVE=0.52	α = 0.86 CR=0.86 AVE=0.61	ω= 0.73 AVE= 0.39	ω= 0.79 AVE=0.48	α = 0.81 AVE=0.67	IN AVE= 0.48	IN AVE= 0.60	IN AVE= 0.52	IN AVE=0.55	α = 0.81 AVE=0.55	α = 0.84 IN	CR=0.88 AVE=0.65	α =0.90 ω=0.91 AVE=0.70	0.97 AVE= 0.90	0.97 AVE= 0.90
7.	0.90	0.88	0.89	0.72	0.68	0.62	0.77	0.85	0.006	0.72	0.81	0.66	0.73		0.69	0.77	0.71	0.70	0.70		0.80	0.85	0.93	0.93

13.	0.83	0.81	0.90	0.88	0.74	0.80	0.87	0.90	0.014	0.72	0.76	0.63	0.68	0.84	0.75	0.86	0.77	0.78	0.78		0.82	0.87	0.94	0.94
5.	0.77	0.78	0.76	0.67	0.82	0.65	0.67	0.83	-0.02	0.75	0.78	0.72	0.80		0.63	0.72	0.69	0.76	0.76		0.80	0.83	0.96	0.96
21.	0.70	0.84	0.82	0.76	0.73	0.91	0.76	0.78	-0.03	0.70	0.79	0.47	0.53	0.80	0.70	0.75	0.72	0.74	0.74		0.85	0.79	0.96	0.96
IM to know	α = 0.91	α = 0.91	α = 0.89		α = 0.78 AVE=0.43				IN AVE=0.38										α = 0.85 AVE=0.68	α = 0.90 IN				
					0.59				0.58										0.77					
					0.75				0.61										0.85					
					0.58				0.66										0.88					
					0.68				0.62										0.79					
IM to experience stimulation	α = 0.78	α = 0.80	α = 0.78		α = 0.78 AVE=0.48				α = 0.92 AVE=0.60										α = 0.80 AVE=0.55	α = 0.94 IN				
					0.58				0.70										0.66					
					0.72				0.83										0.80					
					0.69				0.75										0.75					
					0.76				0.80										0.76					
IM to accomplish	α = 0.80	α = 0.83	α = 0.83		α = 0.80 AVE=0.50				IN AVE=0.52										α = 0.79 AVE=0.57	α = 0.86 IN				
					0.68				0.76										0.76					
					0.72				0.63										0.79					
					0.74				0.72										0.72					
					0.70				0.76										0.76					
Autonomous									α = 0.93 IN															ω=0.90
Controlled							α = 0.89																	
Discriminant validity		Problems in: Integ/ident	Problems in: Intro/ext	Problems in: Intro/ext; Ext/Am	Problems in: IMStimu/IMKnow; IMAccomp/IMKnow; IMStimu/IMAccomp; Ident/IMAccomp; Ident/integer; Ext/Am	Problems in: Ext/intro; Ext/Am	Problems in: Im/integ; Integ/ident	-x-	No data	No data	Problems in: Ext/intro; Ident/integer Integ/Im	Problems in: Im/am; Intro/ext; Intro/am	Problems in: Intro/ext;	Problems in: Ext/Am	No data	No data	No data	Problems in: Intro/Ext; Ext/Am	Problems in: IMAccomp/IMStimu; intro/ext; ext/am	No data	Problems in: Ext/intro; Ident/integer	Problems in: Im/integ; Integ/ident; Intro/ext; Am/ext	Problems in: Ident/integ	-x-

Appendix C

Tables relating to the adjustment and invariance indices for both scales

Table C.1

SMS-II Fit Indices and SMS-II Invariance

	MODELS	CFI	TLI	RMSEA	RMSEA90%CI	SRMR	X2	Degrees of freedom	X2/degrees of freedom	Invariance
Pelletier et al. (2013)	Study 1	0.94	0.92	0.06	0.04-0.06		231.88 p<0.001	120	1.93	
	Study 2	0.94	0.92	0.07	0.05-0.08		258.14 p<0.001	120	2.15	The factor structure of the SMS-II is invariant between men and women (Δx² p>0.05), but the comparison between age groups (under and over 40 years) indicated a significant difference (Δx² p=0.04), which compromised the assumption of invariance between age groups.
Nascimento et al. (2014)	Initial model	0.86	0.83	0.08	0.07-0.09		391.21	120	3.26	
	Alternative model – six correlations between the standard errors obtained with the modification indices were added to the model	0.93	0.91	0.06	0.05-0.07		249.55 p<0.001	112	2.23	The structure of the SMS-II factors for the Portuguese language (Brazil) is invariable between men and women (Δx2, p>0.05). They evaluated and verified temporal stability (7 days)
Stenling et al. (2015)	Original model	0.87		0.08	0.08-0.09	0.08	433.06	120	3.61	No data
Pineda-Espejel, et al (2016)	M1: Original model	0.97	0.96	0.08	0.07-0.09		306.56	120	2.55	No data
	M1A: 6 factors; 17 items (item 16 was eliminated due to its low factorial weight (λ = 0.16, p<0.05); Better than M1	0.98	0.96	0.06	0.05-0.07		202.13	104	1.94	No data
	M2: 3 factors; 17 items (Autonomous; Controlled; Amotivation)	0.97	0.97	0.07	0.06-0.08		258.54	116	2.23	No data
	M2A: 3 factors; 16 items (item 11 was eliminated due to its low factorial weight (λ = 0.34, p<0.01); There are no significant differences between models 2 and 2A	0.98	0.97	0.07	0.06-0.08		221.50	101	2.19	No data
Viciana et al. (2017)	S-SMS-II-2 - autonomous motivation and controlled motivation	0.81	0.78	0.09			697.79		7.84	
	S-SMS-II-3 - autonomous motivation, controlled motivation and amotivation	0.78	0.73	0.09			647.78		4.91	
	S-SMS-II-6	0.93	0.91	0.05			392.66		3.27	
	S-SMS-II-5 - the introjected regulation factor and item 15 were eliminated due to their insufficient discriminant validity	0.97	0.95	0.04			167.38		2.50	
	CFA male - using SMS-II-5	0.97	0.96	0.04			125.89 p<0.01		1.89	The metric invariance of the SMS-II between men and women was not confirmed (Δx2, p<0.05), while the scalar invariance was maintained (Δx2, p>0.05).
	Female CFA - using SMS-II-5	0.93	0.91	0.06			138.07 p<0.01		2.06	
	CFA federated athletes - using SMS-II-5	0.95	0.93	0.05			146.27 p<0.05		2.29	As for the Federated and Non-Federated groups, metric invariance was observed (Δx2, p>0.05), but scalar invariance was not confirmed (Δx2, p<0.05).
	Non-federated CFAs - using SMS-II-5	0.99	0.99	0.03			75.41 p<0.05		1.18	
Paic et al. (2017)	Model 1: 6 factors; 19 items	0.96	0.95	0.05		0.04			2.33	No data
	Model 2: with a latent intrinsic factor that measures issues in different subfactors: intrinsic motivations oriented towards efficiency in psychomotor learning (acquisition of techniques) and cognitive learning in intrinsic motivation.	0.98	0.97	0.04		0.04			1.77	No data
Granero-Gallegos et al. (2018)	6 factors; 18 items	0.94	0.95	0.05	0.05-0.06	0.05	481.57 p<0.001		4.01	The factor structure of the SMS-II is invariant between men and women (ΔCFI<0.01) They assessed and verified temporal stability (4 weeks)
Li et al. (2016)	Study 1: 6 factors; 18 items	0.93	0.91	0.05	0.04-0.07	0.07	214.56 p<0.001	120	1.79	
	Study 2	0.93	0.91	0.05	0.04-0.06	0.06	201.15 p<0.001	120	1.68	The factor structure of the SMS-II is invariant between men and women (ΔCFI<0.01) and Juniors (16–20) and Seniors (21–25)
Vallejo-Reyes et al. (2018)	No confirmatory analysis data									No data
Pelletier et al. (2017)	6 factors; 18 items	0.93	0.91	0.06	0.05-0.07	0.06	230.51 p<0.001	120	1.92	

	Men	0.91	0.90	0.06	0.05-0.07	0.06	188.17 p<0.001	120	1.57	
	Women	0.92	0.90	0.06	0.05-0.07	0.07	239.64 p<0.001	120	2.00	
	CFA model for sex without restrictions	0.92	0.90	0.06	0.05-0.07	0.06	430.59 p<0.001	240	1.79	
	English	0.92	0.90	0.06	0.05-0.07	0.06	241.70 p<0.001	120	2.01	
Chin et al. (2021)	6 factors; 18 items	0.91	0.88	0.06	0.05-0.06	0.06				No data
	Alternative model - correlation between the residuals of items with the same factor	0.92	0.90	0.05	0.05-0.06	0.06				No data
Smohai et al. (2021)	Competitive athletes (N = 718)	0.92	0.89	0.06		0.07				No data
	Recreational athletes (N = 479)	0.94	0.92	0.06		0.06				No data
Jelinek et al. (2021)	6 factors; 18 items	0.91	0.89	0.08	0.06-0.09		255.35 p<0.01	120	2.13	No data
	4 factors; 18 items: autonomous motivation (intrinsic, integrated and identified regulation), introjected, external and amotivation	0.90	0.88	0.08	0.07-0.09		282.14 p<0.01	129	2.19	No data
Rodrigues et al. (2021)	6-factor correlated model - team sports	0.92	0.90	0.08	0.07-0.08	0.06	785.75 p<0.001	120	6.55	The factor structure of the SMS-II is invariant between team and individual sports (ΔCFI<0.01)
	6-Correlated Factor Model - Individual Sports	0.92	0.90	0.08	0.07-0.08	0.06	821.04 p<0.001	120	6.84	
	6-Correlated Factor Model - Men	0.91	0.91	0.07	0.06- 0.08	0.05	1263.46 p<0.001	120	10.53	The factor structure of the SMS-II is invariant between men and women (ΔCFI<0.01)
	6-Correlated Factor Model - Women	0.91	0.90	0.08	0.07-0.08	0.06	961.10 p<0.001	120	8.01	
	6-factor correlated model - total sample	0.91	0.90	0.08	0.07-0.09	0.08	1368.30 p<0.001	120	11.40	
Barreira et al. (2022)	Study 1 - model 1 (n=304)	0.88	0.85	0.09			386.52 p<0.001	120	3.22	No data
	Study 1 - model 2 (modification indices and tracking covariance adjustments) (n=304)	0.94	0.92	0.06			262.19 p<0.001	116	2.26	No data
	Study 2 - model 1 (n=441)	0.85	0.81	0.08			472.32 p<0.001	120	3.94	No data
	Study 2 - model 2 (modification indices and tracking covariance adjustments) (n=441)	0.92	0.90	0.06			298.66 p<0.001	114	2.62	No data
	Study 3 - model 1 (n=310)	0.89	0.86	0.07			319.75 p<0.001	120	2.66	No data
	Study 3 - model 2 (modification indices and tracking covariance adjustments) (n=310)	0.90	0.87	0.07			310.82 p<0.001	118	2.66	No data
Baaziz et al. (2023)	6 factors; 18 items	0.99	0.99	0.02	0.01-0.03	0.02	144.59 p > 0.063	120	1.20	They assessed and verified temporal stability (4 weeks)
Pereira et al. (2024)	6 factors; 18 items	0.90	0.87	0.10		0.09	443.07 p<0.001	239	1.85	
	Alternative model - eliminating 21 observations that turned out to be outliers; including trajectories between the residuals of the pairs of items that included the same factor, namely, 3 and 17 (intrinsic motivation), 6 and 12 (identified regulation), 7 and 16 (introjected regulation), and 2 and 13 (amotivation)	0.95	0.93	0.06		0.05	264.19 p<0.001	239	1.11	The factor structure showed invariance between sexs (Δx² p>0.05) and temporal stability was confirmed after 7 days.

Table C.6

BRSQ Fit Indices and BRSQ Invariance

	MODELS	CFI	TLI	RMSEA	RMSEA90%CI	SRMR	X2	Degrees of freedom	X2/degrees of freedom	Invariance
Lonsdale et al. (2008)	Study 1 – 8 factors; 32 items<br>N=382	0.96	0.96	0.05	0.04–0.05		854.62<br>P<0.01	382	2.24	
	Study 2 – BRSQ6 N=343	0.99	0.99	0.04	0.03-0.05		385.44<br>P<0.01	237	1.63	The structure of both models (BRSQ6 and BRSQ8) were invariant between sexs (ΔCFI<0.01)
	Study 2 – BRSQ8 N=343	0.97	0.97	0.03	0.03-0.05		593.55<br>P<0.01	436	1.36	
	Study 2 - alternative model - created a single controlled EM factor	ΔCFI=-0.02<br>Worse than<br>BRSQ6	IN	IN	IN		240.92<br>P<0.001			
	Study 2 - alternative model - the covariance of the identified and integrated factors was set at 1.0, but the covariance between the external and introjected regulation factors, as well as all other interfactor correlations were estimated	ΔCFI=-0.02<br>Worse than<br>BRSQ6					97.68<br>P<0.001			
	Study 2 - alternative model - the covariances of amotivation, external regulation and introjected regulation were set at 1.0, effectively forming a single factor of non-self-determined motivation	ΔCFI=-0.04<br>Worse than<br>BRSQ6					624.89<br>P<0.001			
	Study 2 - alternative model - the covariances for identified regulation, integrated regulation and intrinsic motivation were set at 1.0, effectively forming a single autonomous motivation factor	ΔCFI=-0.05<br>Worse than<br>BRSQ6					740.87<br>P<0.001			
	Study 3– BRSQ6	0.97	0.97	0.07	0.06-0.08		601.44	237	2.54	
	Study 3– BRSQ8	0.97	0.97	0.06	0.06-0.07		982.15	436	2.25	
Viladrich, et al., (2011)	M1 - extrinsic motivation: external, introjected, identified and integrated regulation	0.69	0.65	0.13			2546.99<br>P<0.001	246	10.35	
	6 factors; 24 items Best-fit model	0.92	0.91	0.07			815.41<br>P<0.001	237	3.44	
	Controlled (external and internalized) and Autonomous (identified and integrated)	0.88	0.87	0.08			1129.16<br>P<0.001	246	4.59	
Moreno-Murcia et al. (2011)	8 factors - 36 items	0.89	0.87	0.05		0.06	967.22			
	8 factors; 32 items - the following errors were correlated: E11-E20 (factor: knowledge of GI), E6-E24 and E6-E33 (identified), E5-E23 (integrated), E16-E34 (introjected), E17-E26 (factor: external) and E8-E17 (external). Best-fit model	0.92	0.92	0.04		0.06	824.56			
Viladrich et al. (2013)	5 factors - no integrated regulation	0.89	0.87	0.08	0.08-0.08		8616.48	800	1.02	The factor structure of the model is invariant across 5 European countries (ΔCFI<0.01)
Shokri et al., (2014)	6 factors	0.94	0.93	0.09			534.05	237	2.25	
	4 factors intrinsic, integrated, identified and controlled regulation	0.93	0.92	0.09			586.61	246	2.38	
Hancox et al. (2015)	6 factors<br>Best fit model	0.95	0.94	0.07	0.06–0.07		1517.90<br>P<0.001	237	6.40	The factor structure of the model is invariant across different levels of dance (recreation; vocation), ages (<18 years; ≥18 years); sex (ΔCFI < 0.01)
	M2:im; autonomous extrinsic; controlled extrinsic; am<br>Good fit, ΔCFI<0.01	0.95<br>ΔCFI=0.00	0.95	0.06			1460.97<br>P<0.001	242	6.04	
	M3: im; extrinsic; AM<br>Adjustment not acceptable	0.84<br>ΔCFI=-0.12	0.81	0.12			4514.57<br>P<0.001	245	18.43	
	M4: autonomous; controlled extrinsic; am<br>Good fit, but the ΔCFI value between M4 and M1>0.01, indicating that, in terms of factorial validity, M1 is preferable	0.93<br>ΔCFI=-0-03	0.92	0.08			2187.10<br>P<0.001	244	8.96	
	M5: IM; IGeID; IJ; EX; AM	0.94<br>ΔCFI=-0.02	0.93	0.08			1890.03<br>P<0.001	242	7.81	
	M6: IM; IG; ID; IJeEX; AM	0.94<br>ΔCFI=-0.01	0.94	0.07			1718.18<br>P<0.001	243	7.07	
Tsitskari et al. (2015)	36 items	0.58	0.56	1.80	0.25-0.27	1.804	6519.16<br>P=1.00	630	10.35	
Çetinkaya and Mutluer, (2018)	6 factors – 24 items	0.97	0.97	0.06			753.78	237	1.20	
Monteiro et al., (2018)	6 factors – 24 items	0.90	0.89	0.07	0.067-0.07	0.07	1308.0<br>P<0.001	237	5.51	

	Since the TLI did not reach the cut-off value, the model was readjusted with the elimination of two items (regulation 11-integrated; regulation 15-introjected) because the items presented higher correlation values with other factors - no improvements were observed, so the elimination was not justified.	0.92	0.90	0.07	0.06-0.07	0.06	995.1<br>P<0.001	194	5.12	
	M3: 2 second-order factors, 6 first-order factors and 24 items	0.88	0.87	0.07	0.07-0.07	0.08	1536.5	257	6.22	
	M4: 2 second-order factors, 5 first-order factors and 20 items (without first-order amotivation)	0.88	0.86	0.08	0.08-0.09	0.09	1164.3	164	7.09	
Stenling et al., (2018)	T1-5 factors - without integrated regulation	0.95	0.94	0.04	0.03-0.05	0.05	260.06	160	1.63	Time invariance tested and proven over a 5-month period (ΔCFI < 0.01)
	T2-5 factors - without integrated regulation	0.92	0.90	0.06	0.05-0.06	0.05	337.41	160	2.10	
Francisco et al., (2019)	6 factors; 12 items	0.97	0.95	0.06	0.04-0.07	0.03	102.08<br>P<0.001	39	2.62	
Cece et al., (2019)	T1	0.94	0.93	0.04	0.03-0.04	0.05	304.98	160	1.91	Temporal invariance tested and verified (ΔCFI<0.01)
	T2	0.93	0.92	0.05	0.04-0.06	0.06	384.30	160	2.40	
	T3	0.94	0.93	0.05	0.04-0.06	0.06	315.06	160	1.97	
Guedes et al., (2019)	9 factors	0.94		0.05	0.04-0.06				1.87	
	6 factors	0.94		0.05	0.04–0.06				1.52	The factor structure of the model is invariant across sexs and ages (14; 15 and 16; 17) (ΔCFI < 0.01)
	8 factors	0.94		0.07	0.06– 0.08				1.96	
Filippos et al. (2019)	9 factors	0.93		0.07		0.04	1528.31			
Monteiro et al. (2019)	General model	0.91	0.90	0.08	0.07–0.08	0.06	2735.18	237	11.54	
	Men	0.91	0.90	0.07	0.07–0.08	0.06	1687.76	237	7.12	The factor structure of the model is invariant across sexs and different types of sports (ΔCFI < 0.01)
	Women	0.91	0.90	0.08	0.08–0.09	0.06	1201.06	237	5.06	
	Soccer	0.91	0.90	0.07	0.06–0.07	0.06	816.41	237	3.44	
	Swimming	0.92	0.90	0.08	0.07–0.09	0.06	1884.38	237	7.95	
	Basketball	0.92	0.90	0.08	0.07–0.09	0.06	703.94	237	2.97	
	Parasport	0.91	0.90	0.08	0.08–0.08	0.06	589.67	237	2.48	
Alexe et al. (2022)	Primary order models: Intrinsic, extrinsic, amotivation	0.66	0.63	0.14	0.13-0.14	0.16	2969.36	249	11.93	
	Primary order models: Autonomous motivation; controlled motivation; amotivation	0.88	0.86	0.08	0.08-0.09	0.06	1241.08	249	4.99	
	Primary order models: Intrinsic; autonomous extrinsic motivation; controlled extrinsic motivation; amotivation	0.89	0.88	0.08	0.07-0.08	0.06	1135.16	246	4.61	
	Primary order models: Intrinsic; integrated and identified regulation;<br>Primary order models: Introjected; externalized; amotivation	0.90	0.88	0.08	0.07-0.08	0.06	1076.35	242	4.45	
	Primary order models: Intrinsic; integrated; identified; Introjected and external; amotivation	0.90	0.89	0.08	0.07-0.08	0.05	1054.92	242	4.36	
	Primary Order Models: 6 Factors	0.93	0.91	0.07	0.06-0.07	0.05	857.96	237	3.62	The factor structure of the model is invariant across different ages (18-21 and 22-52 years) and sports (individual and collective) (ΔCFI < 0.01)
	Hierarchical models: Intrinsic; extrinsic; amotivation	0.81	0.78	0.10	0.09-0.12	0.15	1799.95	245	7.35	
	Hierarchical models: Autonomous; controlled; amotivation	0.92	0.91	0.07	0.06-0.07	0.05	873.55	244	3.58	
Luo et al. (2024)	M1: 6 factors; good fit	0.98	0.97	0.04	0.03–0.05		382.17	237	1.61	
	M2: AM; Controlled; Autonomous	0.78	0.75	0.12	0.12–0.13		1566.67	249	6.29	
	M3: IM; IG; ID; IJ; EX; AM; Controlled; Autonomous; Good fit	0.93	0.93	0.07	0.06–0.08		637.36	232	2.75	
	M4:AM;EX;IJ; Autonomous; Controlled	0.88	0.86	0.09	0.08–0.09		955.45	244	3.92	
	M5:AM; ID; IG; IM; Autonomous and controlled;	0.80	0.77	0.12	0.11–0.12		1409.37	240	5.87	
	M6:AM; Controlled; ID; IG;IM	0.83	0.80	0.12	0.10–0.11		1264.80	242	5.23	
	M7: AM; EX; IT; Autonomous; Good fit	0.92	0.91	0.07	0.07–0.08		719.88	246	2.93	
	Mfinal: AM; EX; IT; Autonomous (without item 12 of the autonomous regulation)	0.98	0.97	0.48	0.04-0.06		180.81	98	1.85	The factor structure of the model is invariant across sex and sporting level (competition; professional) (ΔCFI < 0.01)
	Mfinal men	0.98	0.98	0.04	0.03–0.06		146.99	98		
	Mfinal women	0.93	0.91	0.08	0.06–0.10		157.48	98		
	Competition	0.97	0.96	0.06	0.04–0.08		141.81	98		
	Professional	0.97	0.97	0.05	0.04–0.07		156.05	98		

Appendix D

Tables relating the effect of correlations on the SMS-II and BRSQ motivational continuum

Table D.7

Effect of correlations on the SMS-II and BRSQ motivational continuum

	IM_INTE	IM_IDENT	IM_INTRO	IM_EXT	IMAM	INTE_IDENT	INTE_INTRO	INTE_EXT	INTE_AM	IDENT_INTRO	IDENT_EXT	IDENT_AM	INTRO_EXT	INTRO_AM	EXT_AM
BRSQ	0.74	0.56	-0.24	-0.40	-0.47	0.77	0.03	-0.19	-0.29	0.12	-0.07	-0.19	0.77	0.73	0.73
SMS-II	0.69	0.71	0.40	-0.03	-0.30	0.73	0.54	0.07	-0.29	0.56	0.09	-0.23	0.36	0.49	0.49
